# Supplementary material for: Medicine information helpline after hospitalization–a randomized trial: Impact on patient satisfaction, patient concerns about medicines and clinical outcome on patient safety
Source: PLoS One. 2023 Oct 26;18(10):e0293523. doi: 10.1371/journal.pone.0293523 (PMC10602279; doi:10.1371/journal.pone.0293523)
Supplement: S1 Appendix — (DOCX) [file pone.0293523.s002.docx]

**S2 Appendix:** Interview guide

| **No** | **Question** | **Answer type** | **Group** |
| --- | --- | --- | --- |
| Medicine information during hospitalization and after discharge | | | |
| 1 | Were you satisfied with the medicine information you received while being hospitalized? | Ratings 1-5^a^ | All |
| 2 | Were there any changes in your medication while you were hospitalized? | Yes/No/Do not know | All |
| 3 | Did you receive a medication-status-list when you were discharged from the hospital? | Yes/No/Do not know | All |
| 4 | Were you satisfied with the medicine information you received when you were discharged from the hospital? | Ratings 1-5^a^ | All |
| 5 | How would you like to receive information about your medication and why? | Written/Oral/Free text | All |
| 6 | Have you had any questions regarding your medication after being discharged from the hospital? | Yes/No/Do not know | All |
| 7 | If yes to question 7: Where did you search for information? | Free text | All |
| 8 | If yes to question 7: Have you received an answer to your question? | Yes/No/Do not know | All |
| Beliefs and perception of security about medicines | | | |
| 9 | Have you felt safe about your medication after you were discharged from the hospital? | Ratings 1-5^a^ | All |
| 10 | My health, at present, depends on my medicines. | Ratings 1-5^a^ | All |
| 11 | Having to take medicines worries me. | Ratings 1-5^a^ | All |
| 12 | My medicines are a mystery to me. | Ratings 1-5^a^ | All |
| 13 | My medicines disrupt my life. | Ratings 1-5^a^ | All |
| Patient satisfaction with the MIH | | | |
| 14 | Has the offer to contact the medicine information helpline with questions regarding your medication increased your sense of security after being discharged from the hospital? | Yes/No/Do not know | IG+IGQ |
| 15 | Have you contacted the medicine information helpline after being discharged from the hospital? |  | IG+IGQ |
| 16 | Have you contacted the medicine information helpline by telephone or email? | Telephone/Email | IGQ |
| 17 | Did you receive the answer by telephone or email? | Telephone/Email/Both | IGQ |
| 18 | Was there a timely (immediate) answer to your question? | Immediate/ Within deadline / After deadline | IGQ |
| 19 | Was the answer comprehendible? | Ratings 1-5^a^ | IGQ |
| 20 | Did you receive the information needed? | Ratings 1-5^a^ | IGQ |
| 21 | Did the answer influence your medication consumption? | Ratings 1-5^a^ | IGQ |
| 22 | Are you satisfied with the answer you received? | Ratings 1-5^a^ | IGQ |
| 23 | Have you felt safe regarding your medication after you contacted the MIH? | Ratings 1-5^a^ | IGQ |
| 24 | Describe with your own words, which information from the MIH regarding your medication have been beneficial to you and how? | Free text | IGQ |
| 25 | Are you satisfied with the MIH? | Ratings 1-5^a^ | IGQ |
| 26 | Would you contact the MIH again? | Yes/No/Do not know | IGQ |
| 27 | Should the MIH become a permanent service in the Capital Region Denmark? | Yes/No/Do not know | IG+IGQ |
| ^a^ Ratings: 1 = not at all, 2 = to a small extent, 3 = to some extent, 4 = to a great extent, 5 = to a very great extent.  If the participant rates 1, then he or she will be asked to specify.  All: CG (control group), IG (Intervention group who did not make any enquiries), IGQ (Intervention group who made enquiries) | | | |
