# Supplementary material for: Medicine information helpline after hospitalization–a randomized trial: Impact on patient satisfaction, patient concerns about medicines and clinical outcome on patient safety
Source: PLoS One. 2023 Oct 26;18(10):e0293523. doi: 10.1371/journal.pone.0293523 (PMC10602279; doi:10.1371/journal.pone.0293523)
Supplement: S2 Appendix — (DOCX) [file pone.0293523.s003.docx]

**S3 Appendix:** results based in the interview guide

| **Questions from the interview guide** | **Control group (CG)**  *33 participants* | **Intervention group (IG)**  *119 participants* | **Intervention group who made enquiries before the interview (IGQ)** |  |
| --- | --- | --- | --- | --- |
| Medicine information during hospitalization and after discharge | | | |  |
| 1. Were you satisfied with the medicine information you received while being hospitalized?^a^ | 4.06 ± 1.29 (n=32)  Don’t know: 0  Irrelevant: 1 | 3.91 ± 1.18  (n=109)  Don’t know: 4  Irrelevant: 6 | 3.63 ± 1.30 (n=8) Don’t know: 0  Irrelevant: 0 |  |
| 1. Were there any changes in your medication while you were hospitalized?^b^ | Yes: 18 (55)  No: 11 (33)  Don’t know: 4(12)  (n=33)  Irrelevant: 0 | Yes: 68 (58)  No: 42 (35) Don’t know: 8 (7)  (n=118)  Irrelevant: 1 | Yes: 6 (75)  No: 2 (25)  Don’t know: 0  (n=8)  Irrelevant: 0 |  |
| 1. Did you receive a medication-status-list when you were discharged from the hospital?^b^ | Yes: 25 (76)  No: 7 (21)  Don’t know: 1(3)  (n=33)  Irrelevant: 0 | Yes: 75 (63)  No: 34 (29)  Don’t know: 9(8)  (n=118)  Irrelevant: 1 | Yes: 3 (38)  No: 4 (50)  Don’t know: 1(12)  (n=8)  Irrelevant: 0 |  |
| 1. Were you satisfied with the medicine information you received when you were discharged from the hospital?^a^ | 4.13 ± 1.09 (n=31) Don’t know: 2  Irrelevant: 0 | 3.89 ± 1.32  (n=104) Don’t know: 5  Irrelevant: 10 | 3.43 ± 1.81 (n=7) Don’t know: 0  Irrelevant: 1 |  |
| 1. How would you like to receive information about your medication and why?^c^ | Oral: 5  Written: 13  Oral + written: 13  Other: 1  (n= 32)  Irrelevant: 1 | Oral: 10  Written: 40  Oral + written: 60  Other: 0  (n=110)  Irrelevant: 9 |  |  |
| 1. Have you had any questions regarding your medication after being discharged from the hospital?^b^ | Yes: 6 (18)  No: 27 (82) Don’t know: 0  (n=33)  Irrelevant: 0 | Yes: 33 (28)  No: 83 (71) Don’t know: 1(1)  (n=117)  Irrelevant: 2 | Yes: 8 (100)  No: 0 (0) Don’t know: 0  (n=8)  Irrelevant: 0 |  |
| 1. If yes to question 6: Where did you search for information?^c^ | General practitioner: 2  The internet: 1  Pharmacy: 0  Hospital: 1  Relatives: 1  Acquaintances: 0  District nurse: 1  MIH: 0  Other: 0  No search: 1 | General practitioner: 12  The internet: 2  Pharmacy: 1  Hospital: 2  Relatives: 1  Acquaintances: 0  District nurse: 2  MIH: 8  Other: 1  No search: 9 | General practitioner: 0  The internet: 0  Pharmacy: 1  Hospital: 1  Relatives: 0  Acquaintances: 0  District nurse: 0  MIH: 8  Other: 0  No search: 0 |  |
| 1. If yes to question 7: Have you received an answer to your question?^b^ | Yes: 4 (67)  No: 1 (17)  No search: 1 (17)  (n=6) | Yes: 22 (67)  No: 4 (12)  No search: 7 (21)  (n=33) | Yes: 8 (100)  No: 0 (0)  No search: 0 (0)  (n=8) |  |
| Beliefs and perception of safety about medicines | | | |  |
| 1. Have you felt safe about your medication after you were discharged from the hospital?^a^ | 4.21 ± 0.89 (n=33)  Don’t know: 0  Irrelevant: 0 | 4.03 ± 1.13 (n=112) Don’t know: 0  Irrelevant: 7 | 2.75 ± 1.49 (n=8) Don’t know: 0  Irrelevant: 0 |  |
| 1. My health. at present. depends on my medicines^a^ | 4.19 ± 1.20 (n=32) Don’t know: 0  Irrelevant: 1 | 3.86 ± 1.46 (n=112) Don’t know: 2  Irrelevant: 5 | 3.88 ± 1.36 (n=8) Don’t know: 0  Irrelevant: 0 |  |
| 1. Having to take medicines worries me^a^ | 2.38 ± 1.43 (n=32) Don’t know: 0  Irrelevant: 1 | 2.41 ± 1.47  (n=115) Don’t know: 0  Irrelevant: 4 | 2.63 ± 1.06 (n=8) Don’t know: 0  Irrelevant: 0 |  |
| 1. My medicines are a mystery to me ^a^ | 1.76 ± 1.17 (n=33)  Don’t know: 0  Irrelevant: 0 | 1.90 ± 1.29  (n=113)  Don’t know: 1  Irrelevant: 5 | 2.63 ± 1.51 (n=8) Don’t know: 0  Irrelevant: 0 |  |
| 1. My medicines disrupt my life ^a^ | 2.00 ± 1.34 (n=32)  Don’t know: 0  Irrelevant: 1 | 2.05 ± 1.32 (n=111)  Don’t know: 2  Irrelevant: 6 | 2.33 ± 1.37 (n=6) Don’t know: 2  Irrelevant: 0 |  |
| Patient satisfaction with the MIH | | | |  |
| 1. Has the offer to contact the MIH with questions regarding your medication increased your sense of security after being discharged from the hospital?^b^ |  | Yes: 79 (72)  No: 24 (22) Don’t know: 7(6)  (n=110)  Irrelevant: 9 | Yes: 8 (100)  No: 0 (0) Don’t know: 0  (n=8)  Irrelevant: 0 |  |
| 1. Have you contacted the MIH after being discharged from the hospital?^e^ |  |  | Yes: 8 (7)  No: 111 (93)  (n=119) |  |
| 1. Have you contacted the MIH by telephone or email?^e^ |  |  | Phone: 8 (100)  Mail: 0 (0) (n=8) |  |
| 1. Did you receive the answer by telephone or Email ^e^ |  |  | Phone: 7 (100)  Mail: 0 (0) (n=7) Irrelevant: 1 |  |
| 1. Was there a timely (immediate) answer to your question?^f^ |  |  | Immediate: 7 (100) (n=7) Irrelevant = 1 |  |
| 1. Was the answer comprehensible?^a^ |  |  | 4.86 ± 0.35 (n=7) Irrelevant = 1 |  |
| 1. Did you receive the information needed?^a^ |  |  | 4.71 ± 0.45 (n=7) Irrelevant = 1 |  |
| 1. Did the answer influence your medication consumption?^a^ |  |  | 4.14 ± 1.36 (n=7) Irrelevant = 1 |  |
| 1. Are you satisfied with the answer you received?^a^ |  |  | 4.71 ± 0.45 (n=7) Irrelevant = 1 |  |
| 1. Have you felt safe regarding your medication after you contacted the MIH?^a^ |  |  | 4.57 ± 0.73 (n=7)  Irrelevant = 1 |  |
| 1. Describe with your own words, which information from the MIH regarding your medication have been beneficial to you and how?^c^ |  |  | *Qualitative descriptions* |  |
| 1. Are you satisfied with the MIH?^a^ |  |  | 4.57 ± 0.73 (n=7) Irrelevant = 1 |  |
| 1. Would you contact the MIH again?^b^ |  |  | Yes: 7 (100)  No: 0 (0) Don’t know: 0  (n=8)  Irrelevant: 1 |  |
| 1. Should the MIH become a permanent service in the Capital Region Denmark?^b^ |  | Yes: 110 (93)  No: 2 (2) Don’t know: 6(5)  (n=118)  Irrelevant: 1 | Yes: 7 (88)  No: 0 (0) Don’t know: 1(12)  (n=8)  Irrelevant: 0 |  |
| ^a^ Data represented as mean of rating ± SD. Ratings (ordinal): 1=not at all, 2=to a small extent, 3=to some extent, 4=to a great extent, 5=to a very great extent.  ^b^ Data represented as numbers of Yes / No /don’t know (nominal) (% of total).  ^c^ Free text.  ^d^ Data represented as numbers of written information / oral information (% of total).  ^e^ Data represented as numbers of Telephone / Email / Both (% of total).  ^f^ Data represented as numbers of Immediate/ Within deadline / After deadline. | | | | |
