# Supplementary material for: Medicine information helpline after hospitalization–a randomized trial: Impact on patient satisfaction, patient concerns about medicines and clinical outcome on patient safety
Source: PLoS One. 2023 Oct 26;18(10):e0293523. doi: 10.1371/journal.pone.0293523 (PMC10602279; doi:10.1371/journal.pone.0293523)
Supplement: S1 File — (DOCX) [file pone.0293523.s004.docx]

**Lægemiddelrådgivning på tværs af sundhedsvæsenet –**

**betydning for patientsikkerhed og patienternes livskvalitet**

Et forskningsprojekt mellem:

Region Hovedstadens Apotek,

Klinisk Farmakologisk Afdeling, Bispebjerg Hospital,

Lungemedicins Afdeling L, Bispebjerg Hospital

og

Akutmodtagelsen, Bispebjerg Hospital

Kontaktperson:

Marianne Hald Clemmensen

Farmaceut, PhD

Afdelingsleder, Apotekets Information

Region Hovedstadens Apotek

Bispebjerg Bakke 23, Bygn. 51, 2 sal

2400 København NV

marianne.hald.clemmensen@regionh.dk

Tel: 61 77 77 99

## Formål

Projektets formål er at øge patientenes livskvalitet og tryghed i lægemiddelbehandling efter indlægges ved at tilbyde uvildig lægemiddelrådgivning fra en hospitalsbaseret lægemiddelinformationsenhed. Projektet vil tilbyde samme uvildige lægemiddelrådgivning til sundhedsprofessionelle i primærsektoren med det mål at understøtte en mere patientsikker lægemiddelbehandling ved overgang fra hospital til primærsektor.

## Baggrund:

Flere og flere ældre patienter og borgere har i dag komplekse behandlingsforløb, hvor de stifter bekendtskab med sundhedsvæsenets forskellige sektorer. På de danske hospitaler er antallet af udlagte patienter (behandling i eget hjem), ambulante patienter og accelererede patientforløb i stigende grad en virkelighed, som stiller stadig større krav til behandling af patienterne i eget hjem^1,2^. For ældre borgere er den første tid i hjemmet efter indlæggelse ofte krævende, og mange oplever, at der er sket ændringer i deres medicin under indlæggelse som er med til at skabe forvirring og utryghed ^3^.

Overgang fra hospital til hjem udfordrer patientsikkerheden, og en ny rapport fra Dansk Selskab for Patientsikkerhed understreger, at antallet af tværsektorielle hændelser i Danmark er stigende og at disse hændelser ofte omfatter medicineringsfejl ^1^. For mange patienter vil en indlæggelse resultere i ændringer i deres vanlige medicin og/eller opstart af ny medicin. Studier har vist, at op mod 20 % af alle indlagte patienter oplever genindlæggelse indenfor 30 dage efter udskrivelse^4^, og den hyppigste årsag til utilsigtet hændelse efter udskrivelse skyldes udfordringer med patienternes medicinering ^5,6^. Selvom man i dag allerede gør en stor indsats for at forbedre patientsikkerheden ved sektorovergang, synes der stadig at være et stort potentiale i, at understøtte patienterne i deres lægemiddelbehandling, når de udskrives fra hospitalerne.

**Lægemiddelrådgivning på tværs af sektorer**

En af de indsatser, som har vist gode effekter på patientsikkerhed, patienttilfredshed og patient-tryghed er adgang for borgere og sundhedsprofessionelle til uvildig lægemiddelrådgivning ^7-9^. I dette projekt ønsker vi at tilbyde uvildig lægemiddelrådgivning fra en hospitalsbaseret informationsenhed til borgere og til sundhedsprofessionelle i primærsektoren.

Lægemiddelbehandling er gennem tiden blevet mere og mere kompleks og vi benytter i dag flere og flere avancerede og dyre lægemidler. At holde sig orienteret på ekspertniveau er tidskrævende og stiller store krav til den enkelte sundhedsprofessionelle. Adgang til uvildig lægemiddelrådgivning har vist at skabe stor værdi for både patienter og sundhedsprofessionelle og er en vigtig kilde til at opnå relevant information på et højt fagligt niveau ^10,11^.

Trods den øgede kompleksitet i lægemiddelbehandling udskrives mange ældre patienter i dag til fortsat behandling i eget hjem. En stor udfordring i dette skift er at sikre tilgængelig viden og rådgivning om patienternes lægemiddelbehandling herunder information om håndtering, opbevaring og anvendelse af lægemidlerne. I dag er sygehusapotekernes lægemiddelrådgivning forbeholdt personale ansat på regionens hospitaler. Det betyder, at hverken patienter eller sundhedsprofessionelle i primærsektoren har adgang til samme informationsydelser som på hospitalerne. I Sverige etablerede man i 2013 en national sundheds- og plejerådgivning (Vårdguide) med det primære formål at sikre nem adgang for offentligheden til sundhedsfaglig rådgivning. En analyse af henvendelser fra ældre patienter (80 år eller ældre) til Vårdguide viste, at den hyppigste grund til kontakt var lægemiddelrelaterede spørgsmål^12^. Tilsvarende har en række andre lande herunder England, Tyskland og Norge etableret lægemiddelinformationsenheder, som er tilgængelige for borgere og sundhedsprofessionelle uafhængig af sektor^7,8,13^. I Danmark varetages lægemiddelrådgivning i primærsektoren i høj grad af de private apoteker og hos praktiserende læger ^14^. At rådgive om lægemidler kræver specialistviden, og det kan være en udfordring for de private apoteker og praktiserende læger at rådgive om lægemidler som er sygehusspecifikke. Flere studier har vist, at uvildig lægemiddelrådgivning fra lægemiddelinformationsenheder med base på hospitalerne i høj grad kan understøtte en sikker lægemiddelbehandling, understøtte komplians og skabe værdi og tryghed for borgerne ^7-9^. Foruden høj specialviden har lægemiddelinformationsenheder med base på hospitalerne mulighed for hurtig kontakt til de behandlende afdelinger samt mulighed for at orientere sig omkring den enkelte patients indlæggelse hvilket er essentiel for at kunne give lægemiddelrådgivning af høj kvalitet og værdi ^9^.

Som de første i Danmark ønsker vi i dette projekt at etablere en uvildig lægemiddelrådgivning tilknyttet Region Hovedstadens Apotek, som er åben for patienter udskrevet fra udvalgte afdelinger samt for sundhedsprofessionelle ansat på plejecentre i hospitalet optageområde. Vi vil belyse effekten af denne ydelse for patienter og sundhedsprofessionelle, og vi vil som de første også inkludere en kontrolgruppe, som muliggør belysning af ydelsens reelle værdi.

I dette studie fravælges hårde endepunkter som genindlæggelse og utilsigtede hændelser. Mange studier har undersøgt forskellige kliniske farmaceutiske interventioners effekt på netop disse hårde endepunkter, men kun ganske få studier har kunnet vise en signifikant effekt. At måle på disse parametre er meget ressourcekrævende, og det er kendt, at der i et komplekst sundhedssystem er mange faktorer, som kan have betydende indflydelse på disse parametre, og som vi ikke kan kontrollere for ^4^.

## Metode og design

Apotekets Information, Region Hovedstadens Apotek driver i dag en lægemiddelinformationsenhed i samarbejde med Klinisk Farmakologisk Afdeling, Bispebjerg- og Frederiksberg Hospitaler. Lægemiddelinformationsenheden, MedicinInfo, er en veletableret funktion med kvalitetssystemer, databaser til registrering af alle henvendelser samt adgang til relevant litteratur, databaser og opslagsværker. Den grundlæggende struktur for at tilbyde lægemiddelinformation til patienter og sundhedsprofessionelle er derfor etableret. MedicinInfo er åben i apotekets åbningstid og er dagligt bemandet af tre farmaceuter og en læge. Der vil i dette projekt blive rådgivet omkring lægemiddelrelaterede problemstillinger som f.eks. dosering, administration, opbevaring og holdbarhed, produktinformation og identifikation. Hvor det vurderes relevant som f.eks. ved spørgsmål til dosis, specifikt valg af behandling eller lign. vil der blive henvist til anden relevant sundhedsfaglig person (hospitalsafdeling, praktiserende læge eller anden behandler). I dette projekt tilbydes en ydelse hvor patienter og sundhedsprofessionelle kan henvende sig telefonisk i apotekets åbningstid eller kan sende spørgsmål pr. mail. Svar på en henvendelse kan ske mundtligt eller skriftligt.

**Målgruppe:** Projektets primære målgruppe er ældre borgere i medicinsk behandling.

I studie 1 inkluderes patienter/borgere som har været indlagt på enten Lungemedicinsk afdeling eller Akutmodtagelsen på Bispebjerg Hospital. På Lungemedicinsk afdeling udgør andelen af ældre patienter over 75 år omkring 40% mens det estimeres at være lidt højere i Akutmodtagelsen.

Studie 2 er målrettet de sundhedsprofessionelle som skal tage sig af patienten/borgeren efter udskrivelse fra et hospital. Projektet vil tage udgangspunkt i plejecentre som ligger lokalt i forhold til Bispebjerg Hospital. I første omgang inkluderes Plejecenter Poppelbo og Plejecenter Lærkebo.

**Studie 1: Lægemiddelrådgivning til patienter, der udskrives fra et hospitalsophold i Region Hovedstaden**

Interventionsgruppe: Patienter udskrevet fra afdeling L eller Akut-modtagelse på Bispebjerg Hospital

Evaluering: livskvalitet, tryghed i lm. behandling og tilfredshed med information

Kontrolgruppe: Patienter udskrevet fra afdeling L eller Akutmodtagelsen på Bispebjerg Hospital

Evaluering: livskvalitet, tryghed i lm. behandling og tilfredshed med information

Tilbud om lm. rådgivning

Henvendelse om lm. information

I studie 1 tilbydes uvildig lægemiddelrådgivning direkte til patienter udskrevet fra Lungemedicinsk afdeling eller Akutmodtagelsen på Bispebjerg Hospital. Patienter i interventionsgruppen vil få tilbud om uvildig lægemiddelrådgivning fra Region Hovedstadens Apotek, mens patienter i kontrolgruppen får tilbud om information efter vanlig procedure fra den afdeling de udskrives fra.

I studie 1 anvendes et kontrolleret randomiseret design hvor patienterne inkluderes i blokke af 10. Patienter inkluderes ved forespørgsel samt indsamling af skriftlig samtykkeerklæring ved udskrivelse.

De primære endepunkter i studie 1 er patienternes vurdering af livskvalitet, tryghed i lægemiddelbehandling og tilfredshed med lægemiddelinformation. Data for de primære endepunkter vil blive indsamlet gennem struktureret telefoninterview som foretages 10-14 dage efter udskrivelse. Interviewguide udarbejdes som et semi-struktureret spørgeskema og vil tage udgangspunkt i allerede validerede spørgeskemaer ^8,9^. Spørgsmål om livskvalitet vil tage udgangspunkt i EQ-5D.

Data indsamlet fra struktureret interview vil være semi-kvantitative. Et estimat af studiets størrelse gøres på baggrund af scoring af ”tilfredshed med den givne lægemiddelinformation”. Med udgangspunkt i litteraturen estimeres det, at der på en 5 punkts skala opnås en middelværdi på 4,6 +/- 0,7 ^7^. Der findes ingen publicerede resultater som inkluderer baseline målinger, men vi ønsker at kunne detektere en 10 % forbedring i score. I studiet ønskes en power på 90 % og et signifikansniveau på 0,05. Under disse forudsætninger beregnes stikprøvestørrelsen til 48. Det estimeres, at vi med inklusion af patienter udskrevet fra de to afdelinger vil kunne indsamle tilstrækkelig data i den planlagte interventionsperiode på 18 måneder, til at nå en gruppestørrelse på minimum 48.

Foruden primære endepunkter indsamles en række procesdata som bl.a. inkluderer demografiske data, patienternes medicinstatus samt type af henvendelse/spørgsmålskategori.

**Studie 2: Lægemiddelrådgivning til sundhedsprofessionelle i primærsektoren**

Tilbud om lm. rådgivning

Henvendelse om lm. information

Evaluering: Tilfredshed, betydning og kvalitet af lm. information

Interventionsgruppe: Sundhedsprofessionelle i primærsektoren

I studie 2 tilbydes uvildig lægemiddelrådgivning til sundhedsprofessionelle som varetager daglig pleje af ældre borgere i primærsektoren. Vi starter med at inkludere et mindre antal lokale plejecentre med det mål at inkludere flere centre undervejs i studiet, når de første erfaringer er indsamlet. I studie 2 vil sundhedsprofessionelle på Plejecenter Poppelbo og Plejecenter Lærkebo i Københavns Kommune få tilbud om uvildig lægemiddelrådgivning fra Region Hovedstadens Apotek.

I studie 2 anvendes et deskriptivt studiedesign. Studie 2 vil have fokus på at evaluere tilfredsheden med den specifikke ydelse og studiet vil ikke inkludere en kontrolgruppe.

Studie 2 primære endepunkter er sundhedsprofessionelles tilfredshed med den lægemiddelinformation de har modtaget, vurdering af hvilken betydning den givne lægemiddelinformation har for deres arbejde, hvordan de vurderer kvaliteten af den lægemiddelinformation de har fået, samt hvorvidt de har fået lægemiddelinformationen i tide til at kunne benytte den i deres videre arbejde. Data vil blive indsamlet gennem online spørgeskema som sendes til spørger efter de har modtaget svar på henvendelsen. Spørgeskema vil tage udgangspunkt i allerede validerede spørgeskemaer^7,10,11^.

Procesdata i studie 2 inkluderer bl.a. type af henvendelse/spørgsmålskategori, involveret lægemiddel samt registrering af om henvendelse er relateret til hospitalsindlæggelse.

### Databehandling

Data vil blive behandlet i Excel og analyseret med deskriptiv statistik og simpel parametrisk statistik.

## Etik

Studiet vil blive forelagt De Videnskabsetiske Komiteer for Region Hovedstaden samt Styrelsen for Patientsikkerhed. Studiet anmeldes til Datatilsynet og registreres på Current Controlled Trials Ltd.

Der vil blive indsamlet informeret samtykke fra alle inkluderede patienter. Der vil ikke blive rådgivet om patientspecifikke spørgsmål ved henvendelse fra sundhedsprofessionelle i primærsektoren med mindre der er indhentet skriftligt samtykke fra den pågældende patient.

## Inddragelse af patienter

Patienter ønsker at blive involveret og tage ansvar – også i deres lægemiddelbehandling^15^. En større undersøgelse har vist, at netop mulighed for at stille spørgsmål til sundhedspersonale på mail og telefon samt være informeret om, hvem man kan ringe til efter udskrivelse fra sygehus er højt prioriteret blandt danske patienter^16^. I dette projekt er inddragelse af patienten helt centralt idet lægemiddelrådgivningen er målrettet patienten, og det er patienternes evaluering som bruges til at vurdere hvorvidt ydelsen skaber værdi. Vurderes ydelsen af patienterne som værdifuld vil det skabe grundlag for videre implementering af ydelsen.

## Perspektivering

I dette projekt ønsker vi at starte småt, og ydelsen vil i første omgang blive afprøvet i Region Hovedstaden. Vi ønsker dels at få viden om ydelsens værdi, men også om hvilket format som passer bedst ind i et dansk sundhedsvæsen. Med baggrund i disse data vil der være skabt et vigtigt fundament for at afgøre, om ydelsen er relevant at tilbyde fremadrettet til alle patienter og sundhedsprofessionelle i Region Hovedstaden, og om det eventuelt vil være relevant at tilbyde den på nationalt plan.

## Formidlingsplan

Resultaterne fra studiet forventes at resulterer i mindst to videnskabelige publikationer, som vil blive publiceret på engelsk i internationale peer-reviewed tidsskrifter. Projektet vil desuden blive præsenteret på nationale og internationale konferencer.

Data vil blive analyseret med henblik på at identificere om der er spørgsmål/problemstillinger som forekommer med større hyppighed. På den baggrund vil der blive udarbejdet målrettet uddannelsesmateriale, informationsmateriale og kampagner.

## Organisering

Projektet vil blive gennemført som et samarbejde mellem Region Hovedstadens Apotek (Apotekets Information), Lungemedicinsk Afdeling L og Akutmodtagelsen på Bispebjerg Hospital. Der vil blive nedsat en styregruppe for projektet, som har til formål at følge og sikre fremdriften i projektet samt give videnskabelig sparring på projektet. Styregruppen består af: Enhedschef Helle Byg Armandi (Region Hovedstadens Apotek), Afdelingsleder Marianne Hald Clemmensen (PhD, Region Hovedstadens Apotek), Afdelingsleder Charlotte Vermehren (PhD, Klinisk Farmakologisk Afdeling, Bispebjerg Hospital), Afdelingslæge Ditte Skovgaard March (Akutmodtagelsen, Bispebjerg Hospital), Overlæge Lars Pedersen (Lungemedicinsk Afdeling, Bispebjerg Hospital).

Projektet ledes af afdelingsleder Marianne Hald Clemmensen og farmaceut Karianne Willhelmsen Fjære (Region Hovedstadens Apotekets). Lægemiddelrådgivning gives af farmaceuter ansat i Apotekets Information, Region Hovedstadens Apotek.

## Referencer

1. Patientsikkerhed DSf. Patientsikkerhed i det nære og sammenhængende sundhedsvæsen2017.

2. Regioner D. Pres på Sundhedsvæsenet2015.

3. Enheden for Evaluering og Brugerinddragelse RH. Større tryghed i udskrivelsen - patientrejsen fra indlæggelse til udskrivelse: Region Hovedstaden; 2017.

4. Rennke S, Nguyen OK, Shoeb MH, Magan Y, Wachter RM, Ranji SR. Hospital-initiated transitional care interventions as a patient safety strategy: a systematic review. Ann Intern Med 2013;158:433-40.

5. Forster AJ, Murff HJ, Peterson JF, Gandhi TK, Bates DW. The incidence and severity of adverse events affecting patients after discharge from the hospital. Ann Intern Med 2003;138:161-7.

6. Croft LD, Liquori ME, Ladd J, et al. Frequency of Adverse Events Before, During, and After Hospital Admission. South Med J 2016;109:631-5.

7. Bertsche T, Hammerlein A, Schulz M. German national drug information service: user satisfaction and potential positive patient outcomes. Pharm World Sci 2007;29:167-72.

8. Marvin V, Park C, Vaughan L, Valentine J. Phone calls to a hospital medicines information helpline: analysis of queries from members of the public and assessment of potential for harm from their medicines. Int J Pharm Pract 2011;19:115-22.

9. Badiani A WS, Owen S, Parker J, Hall J. Impact of a medicines helpline for patients. European Journal of Hospital Pharmacy 2017:196-9.

10. McEntee JE, Henderson SL, Rutter PM, Rutter J, Davis HJ. Utility and value of a medicines information service provided by pharmacists: a survey of health professionals. Int J Pharm Pract 2010;18:353-61.

11. Bramley DM, Innes AJ, Duggan C, Oborne CA. The impact of Medicines Information enquiry answering on patient care and outcomes. Int J Pharm Pract 2013;21:393-404.

12. Dahlgren K, Holzmann MJ, Carlsson AC, Wandell P, Hasselstrom J, Ruge T. The use of a Swedish telephone medical advice service by the elderly - a population-based study. Scand J Prim Health Care 2017;35:98-104.

13. Schjott J. Benefits of a national network of drug information centres: RELIS. Eur J Clin Pharmacol 2017;73:125-6.

14. Svensberg K, Sporrong SK, Bjornsdottir I. A review of countries' pharmacist-patient communication legal requirements on prescription medications and alignment with practice: Comparison of Nordic countries. Res Social Adm Pharm 2015;11:784-802.

15. Sundhedsvæsenet VfBi. Patientinddragelse i lægemiddelbehandling2016.

16. Patientsikkerhed DSf. Det patientcentrerede sygehus2016.
