# Supplementary material for: Medicine information helpline after hospitalization–a randomized trial: Impact on patient satisfaction, patient concerns about medicines and clinical outcome on patient safety
Source: PLoS One. 2023 Oct 26;18(10):e0293523. doi: 10.1371/journal.pone.0293523 (PMC10602279; doi:10.1371/journal.pone.0293523)
Supplement: S2 File — (DOCX) [file pone.0293523.s005.docx]

**Medicine Information across the healthcare system –**

**impact for patient safety and patients' quality of life**

A research project between:

The Capital Region Pharmacy,

Department of Clinical Pharmacology, Bispebjerg Hospital,

Respiratory Department, Bispebjerg Hospital

and

The Emergency Department, Bispebjerg Hospital

Contact person:

Marianne Hald Clemmensen

Pharmacist, PhD

Head of Department, The Medicine Information Centre

The Capital Region Pharmacy of Denmark

Bispebjerg Bakke 23, Building. 51, 2 sal

2400 Copenhagen NV

marianne.hald.clemmensen@regionh.dk

Phone: 61 77 77 99

**Purpose:**

The aim of the present project is to increase patients' quality of life and safety in their medical treatment after discharge from hospital by offering medicine information from a hospital-based medicine information centre. The project will offer the same independent medicine information to healthcare professionals in the primary sector with the aim of supporting more patient-safe pharmaceutical treatment when transitioning from hospital to the primary sector.

**Background:**

Today more and more elderly patients and citizens are going through complex treatment regimes, where they get in contact with the different sectors of the healthcare system. In Denmark, the number of outpatients and accelerated patient hospitalizations are increasing, which requires greater demands of the treatment of patients in their private homes (1,2). For elderly citizens, the first time at home after hospitalization is often demanding. Many find that there have been changes in their medication during hospitalization, which can lead to confusion and insecurity (3).

Transition from hospital to private home challenges patient safety. A new report from the Danish Society for Patient Safety emphasizes that the number of errors occurring when patients change from hospital to outpatient treatment is increasing and many of these incidents are medication errors (1). For many patients, hospitalization will result in changes to their usual medication and/or starting new medication. Studies have shown that up to 20% of all hospitalized patients experience readmission within 30 days of discharge (4), and that the most frequent cause of unintended events after discharge is due to challenges with the patients' medication (5,6). Although a great effort already has been made today to improve patient safety during healthcare sector transitions, there still seems to be a great potential in supporting patients in their drug treatment when they are discharged from hospitals.

**Medicine information across sectors**

One of the initiatives that has shown a good effect on patient safety, patient satisfaction and patient security is access for citizens and healthcare professionals to independent medicine information (7-9). In this project, we want to offer independent medicine information from a hospital-based information center to citizens and to healthcare professionals in the primary sector.

Medical treatment regimens have become more and more complex in recent years. We use more and more advanced and expensive medicine than before. Keeping informed at an expert level is time-consuming and places great demands on the individual healthcare professional. Access to independent medicine information has been shown to create great value for both patients and healthcare professionals and is an important source for obtaining relevant information at a high professional level (10,11).

Despite the increased complexity of drug treatment, many elderly patients are now a days discharged for continued treatment in their private homes. A major challenge to this change is to ensure available knowledge and information on the patients' drug treatment, including information concerning handling, storage and usage of the drugs. Today, the hospital pharmacies' medicine information is reserved for staff employed at the public hospitals. This means that neither patients nor health professionals in the primary healthcare sector have access to the same information services as in hospitals. In Sweden, a national health and care center (Vårdguide) was established in 2013 with the primary aim of ensuring easy access for the public to healthcare information. An analysis of inquiries from elderly patients (80 years or older) to Vårdguide showed that the most frequent reason for contact was drug-related questions (12). Similarly, a number of other countries, including England, Germany and Norway, have established medicine information centers which are accessible to citizens and healthcare professionals regardless of sector (7,8,13). In Denmark, medicine information in the primary healthcare sector is largely provided by private pharmacies and general practitioners (14). Medicine information requires specialist knowledge, and it can be a challenge for private pharmacies and general practitioners to counsel on hospital-specific medicines. Several studies have shown that independent medicine information from medicine information centers based in hospitals can greatly support safe drug treatment, support compliance and create value and security for citizens (7-9).

In addition to high specialist knowledge, medicine information centers based in the hospitals have the possibility of fast and accessible contact with the treating departments as well as the possibility of orienting themselves around the individual patient's hospitalization, which is essential to provide medicine information of high quality and value (9).

In this project, as the first in Denmark, we want to establish an independent medicine helpline linked to the Capital Region Pharmacy, which is open to patients discharged from selected departments, as well as to healthcare professionals employed at care centers in the hospital's admission area. We will elucidate the effect of this service for patients and healthcare professionals, and we will also be the first to include a control group, which enables elucidation of the real value of the service.

In this study, hard endpoints such as readmission and adverse events are excluded. Many studies have investigated the effect of various clinical pharmaceutical interventions on precisely these hard endpoints, but only very few studies have been able to show a significant effect. Measuring these parameters is very resource-demanding, and it is known that in a complex healthcare system there are many factors that can have a significant influence on these parameters and for which we cannot control (4).

**Method and design**

The Medicines Information Centre (MIC) at The Hospital Pharmacy in the Capital Region of Denmark currently runs a medicines information center in collaboration with the Department of Clinical Pharmacology, Bispebjerg and Frederiksberg Hospitals. The MIC is a well-established department with quality systems, databases for recording all inquiries and has access to relevant literature, databases and reference works. The basic structure for offering medicine information to patients and healthcare professionals is therefore established. The MIC is open during the pharmacy's opening hours on weekdays and is staffed by three pharmacists and a medical doctor. In this project, information will be given on medicines-related issues such as dosage, administration, storage and shelf life, product information and identification. Should inquiries concerning dosage, specific choice of treatment or the like arise, the inquirer will be referred to another relevant healthcare professional (hospital department, general practitioner or other practitioner). In this project, a service is offered where patients and healthcare professionals can contact the helpline by telephone during the hospital pharmacy’s opening hours or can send questions by mail. Responses to inquiries can be made orally or in writing.

**Target group:**
The project's primary target group is elderly citizens undergoing medical treatment.

In study 1, patients/citizens who have been admitted to either the Respiratory Department or the Emergency Department at Bispebjerg Hospital are included. In the Respiratory Department, the proportion of elderly patients over 75 years old is around 40%, while it is estimated to be slightly higher in the Emergency Department.

Study 2 is targeted at healthcare professionals who take care of the patient/citizen after hospital discharge. The project will be introduced at care centers located locally in relation to Bispebjerg Hospital. Initially, the Poppelbo care centre and the Lærkebo care centre will be included.

**Study 1: Medical information for patients being discharged from a hospital stay in the Capital Region of Denmark**

Evaluation: Quality of life, safety in medical treatment and satisfaction with the medicine information

Control group: Patients discharged from the Respiratory Department or the Emergency Department at Bispebjerg Hospital

Evaluation: Quality of life, safety in medical treatment and satisfaction with the medicine information

Intervention group: Patients discharged from the Respiratory Department or the Emergency Department at Bispebjerg Hospital

Inquiry of medicine information

Proposal of medicine information

In study 1, independent medicine information is offered directly to patients discharged from the Respiratory Department or the Emergency Department at Bispebjerg Hospital. Patients in the intervention group will be offered independent medicine information from the Capital Region Pharmacy, while patients in the control group will be offered information according to standard procedure from the department they are discharged from.

In study 1, a controlled randomized design is used where the patients are included in blocks of 10. Patients are included by inquiry and collection of written consent when discharged.

The primary endpoints in study 1 are the patients' assessment of quality of life, safety in medical treatment and satisfaction with medicine information. Data for the primary endpoints will be collected through structured telephone interviews conducted 10-14 days after discharge. The interview guide is prepared as a semi-structured questionnaire and is based on already validated questionnaires (8,9). Questions about quality of life will be based on EQ-5D.

Data collected from the structured interviews will be semi-quantitative. An estimate of the size of the study is made on the basis of scoring of "satisfaction with the given medicine information". Based on the literature, it is estimated that, on a 5-point scale, a mean value of 4.6 +/- 0.77 is achieved. There are no published results that include a baseline measurement. We want to be able to detect a 10% improvement in score. In the study, a power of 90% and a significance level of 0.05 is desired. Under these assumptions, the sample size is calculated to be 48. It is estimated that, with the inclusion of patients discharged from the two departments, we will be able to collect sufficient data during the planned intervention period of 18 months, to reach a group size of at least 48.

In addition to primary endpoints, a number of process data will be collected such as: demographic data, patients' medication status and the type of inquiry/question category.

**Study 2: Medicine information to primary healthcare professionals**

Proposal of medicine information

Inquiry of medicine information

Evaluation: Satisfaction, significance and quality with the medicine information

Intervention group: Primary healthcare professionals

In study 2, independent medicine information is offered to primary healthcare professionals who take care of the elderly citizens. We start by including a small number of local care centers with the aim of including more centers during the study, once the first experiences have been collected. In study 2, healthcare professionals at Care Centre Poppelbo and Care Centre Lærkebo in the Municipality of Copenhagen will be offered independent medicine information from the Capital Region Pharmacy.

In study 2, a descriptive study design is used. Study 2 will focus on evaluating the satisfaction with the specific service and the study will not include a control group.

Study 2 primary endpoints are healthcare professionals' satisfaction with the medicine information they have received, assessment of the significance of the given medicine information for their work, how they assess the quality of the medicine information they have received, and whether they have received the information in time to be able to use it in their further work. Data will be collected through an online questionnaire which is sent to the inquirer after they have received the response to their inquiry. The questionnaire will be based on already validated questionnaires (7,10,11).

Process data in study 2 includes i.a. type of enquiry/question category, medicine involved and registration of whether the inquiry is related to a patient’s hospitalization.

**Data processing**

Data will be processed in Excel and analyzed with descriptive statistics and simple parametric statistics.

**Ethics**

The study will be submitted to the Scientific Ethics Committees for the Capital Region and the Agency for Patient Safety. The study is reported to the Danish Data Protection Authority and registered at Current Controlled Trials Ltd.

Informed consent will be collected from all included patients. Information will not be given on patient-specific questions when inquired by healthcare professionals in the primary sector unless a written consent has been obtained from the patient in question.

**Patient Involvement**

Patients want to be involved and take responsibility – also in their drug treatment (15). A major study has shown that precisely the opportunity to ask questions to healthcare personnel by email and telephone and to be informed about who to call after discharge from hospital is a high priority among Danish patients (16). In this project, the involvement of the patient is absolutely central, as the medicine counseling is targeted at the patient, and it is the patients' evaluation that is used to assess whether the service creates value. If the service is assessed by the patients as valuable, it will create a basis for further implementation of the service.

**Perspectives**

In this project the service will initially be tested in the Capital Region. We partly want to gain knowledge about the value of the service, but also about which format fits best in a Danish healthcare system. Based on this data, an important foundation will be created to determine whether it is relevant to offer the service in the future to all patients and healthcare professionals in the Capital Region, and whether it could be relevant to offer on a national level.

**Dissemination plan**

The results from the study are expected to result in at least two scientific publications, which will be published in English in international peer-reviewed journals. The project will also be presented at national and international conferences.

Data will be further be analyzed with the aim of identifying if there are questions/problems posed to the MIC that occur with greater frequency. Based on this, targeted training material, information material and campaigns will be prepared.

**Organization**

The project will be performed as a collaboration between The Hospital Pharmacy, The Capital Region of Denmark, The Respiratory Department and the Emergency Department at Bispebjerg Hospital. A steering committee will be set up for the project, whose purpose is to monitor and ensure the progress of the project and to provide scientific sparring on the project. The steering group consists of: Head of the Unit Helle Byg Armandi (The Hospital Pharmacy, The Capital Region of Denmark), Head of Department, PhD Marianne Hald Clemmensen (The Hospital Pharmacy, The Capital Region of Denmark), Head of Department, PhD Charlotte Vermehren (Department of Clinical Pharmacology, Bispebjerg Hospital), Department Doctor Ditte Skovgaard March (Emergency Department, Bispebjerg Hospital), Senior physician Lars Pedersen (Department of Respiratory Medicine, Bispebjerg Hospital).

The project is led by department manager Marianne Hald Clemmensen and pharmacist Karianne Willhelmsen Fjaere (The Hospital Pharmacy, The Capital Region of Denmark). Medicine information is provided by pharmacists employed by The Medicine Information Centre, The Hospital Pharmacy at the Capital Region of Denmark.

**Referencer**

1.Patientsikkerhed DSf. Patientsikkerhed i det nære og sammenhængende sundhedsvæsen 2017.

2.Regioner D. Pres på Sundhedsvæsenet 2015.

3.Enheden for Evaluering og Brugerinddragelse RH. Større tryghed i udskrivelsen - patientrejsen fra indlæggelse til udskrivelse: Region Hovedstaden; 2017.

4.Rennke S, Nguyen OK, Shoeb MH, Magan Y, Wachter RM, Ranji SR. Hospital-initiated transitional care interventions as a patient safety strategy: a systematic review. Ann Intern Med 2013;158:433-40.

5.Forster AJ, Murff HJ, Peterson JF, Gandhi TK, Bates DW. The incidence and severity of adverse events affecting patients after discharge from the hospital. Ann Intern Med 2003;138:161-7.

6.Croft LD, Liquori ME, Ladd J, et al. Frequency of Adverse Events Before, During, and After Hospital Admission. South Med J 2016;109:631-5.

7.Bertsche T, Hammerlein A, Schulz M. German national drug information service: user satisfaction and potential positive patient outcomes. Pharm World Sci 2007;29:167-72.

8.Marvin V, Park C, Vaughan L, Valentine J. Phone calls to a hospital medicines information helpline: analysis of queries from members of the public and assessment of potential for harm from their medicines. Int J Pharm Pract 2011;19:115-22.

9.Badiani A WS, Owen S, Parker J, Hall J. Impact of a medicines helpline for patients. European Journal of Hospital Pharmacy 2017:196-9.

10.McEntee JE, Henderson SL, Rutter PM, Rutter J, Davis HJ. Utility and value of a medicines information service provided by pharmacists: a survey of health professionals. Int J Pharm Pract 2010;18:353-61.

11.Bramley DM, Innes AJ, Duggan C, Oborne CA. The impact of Medicines Information enquiry answering on patient care and outcomes. Int J Pharm Pract 2013;21:393-404.

12.Dahlgren K, Holzmann MJ, Carlsson AC, Wandell P, Hasselstrom J, Ruge T. The use of a Swedish telephone medical advice service by the elderly - a population-based study. Scand J Prim Health Care 2017;35:98-104.

13.Schjott J. Benefits of a national network of drug information centres: RELIS. Eur J Clin Pharmacol 2017;73:125-6.

14.Svensberg K, Sporrong SK, Bjornsdottir I. A review of countries' pharmacist-patient communication legal requirements on prescription medications and alignment with practice: Comparison of Nordic countries. Res Social Adm Pharm 2015;11:784-802.

15.Sundhedsvæsenet VfBi. Patientinddragelse i lægemiddelbehandling 2016.

16. Patientsikkerhed DSf. Det patientcentrerede sygehus 2016.
