# Supplementary material for: Medicine information helpline after hospitalization–a randomized trial: Impact on patient satisfaction, patient concerns about medicines and clinical outcome on patient safety
Source: PLoS One. 2023 Oct 26;18(10):e0293523. doi: 10.1371/journal.pone.0293523 (PMC10602279; doi:10.1371/journal.pone.0293523)
Supplement: S3 File — (DOCX) [file pone.0293523.s006.docx]

**S6 Supporting Information:** Raw data from MIH requests

| **Patient**  **number** | **Question** | **Answer** |
| --- | --- | --- |
| 6 | ​Can I take my regular medicine, tablets, today 0.5-1 hour later than the time I usually take my medicine? I usually take at the same time every day, but today I came too early. | ​Yes, you can take your medicine 0.5-1 hour later than the time you usually take your medicine. The medicine you are taking does not lose its effect if the time is changed within the specified time interval. |
| 7 | I was discharged from the hospital yesterday . I was given 2 medicines . I think I get heart palpitations after I take the tablets, 15 minutes after I take them. The palpitations persist a bit and I don't think I've had them before. I don't remember which medicine it is.  Is everything as it should be or do I need to do something?  Can I still drink coffee when I am being treated with the two medicines? | The question is discussed with the doctor, who looks the patient up in SP.  According to the SP and the discharge note, the following has been prescribed for the patient: Prednisolone 25 mg x 1 for 4 days and Cetirizine 10 mg x 1 for 4 days [1].  Palpitations are not a stated side effect of either Prednisolone or Cetirizine [2].  No interactions between the two preparations are reported [3].  The doctor was not worried about further treatment and recommends that the patient continue for the 4 days. If the palpitations persist , it will be relevant for the patient to be investigated for this [4].  The above is communicated to the patient who settles down and will continue with the treatment.  The patient can continue to drink coffee [3]. |
| 8 | The patient wants to speak to the doctor, but has called the TrygMedicin number | Direct the patient to call directly to the nursing home / transfer, as I cannot transfer from the TrygMedicintelefonen . |
| 11 | ​I was discharged yesterday where I took home Primcillin and Propylthiouracil .  Now I have a headache. Can I take the Treo dispersible tablets .? | \| ​The combination can be used. \| ​ \| \| --- \| --- \| |
| 11 | I have been diagnosed with a high metabolism (Graves), and receive 1 tablet daily morning and evening of propylthiouracil .  In addition, I take 1 pill of certirizine daily (for hives) and 1 tablet of multi -tab calcium with vitamin D (500mg).    I have now found some pills to help with thicker hair, but it says in the manual for the pills that you have to check whether you can take them if you take regular medication.    My question is therefore the following;    Can I safely take the pills alongside my regular medication? | It seems to be okay to supplement with the aforementioned food supplement, as long as the daily recommended dosage of vitamins and minerals is not exceeded, which is not the case in this situation. |
| 14 | I was hospitalized with stomach pains. It turned out that I had kidney-pelvic inflammation.  I have now been discharged for 2 weeks and the pain has returned.  I have been referred to the outpatient clinic for follow-up, but not until September 5.  What should I do? The attacks are the same as before I was hospitalized. I'm concerned. | It is considered that this is a medical issue. Perhaps the patient has had a relapse or has not been adequately treated.  The patient says that she had spoken to the doctor when she was hospitalized.  The patient is referred to ward MMA1 and 2, and the telephone number for the department is given. |
| 17 | I have received a prescription for B- combin strong x 3 daily and Thiamin SAD x 1 daily.  I have been to the pharmacy to buy the medicine, but have come home with 2 x Apovit B- combin . What has happened? Can you help? | Both the prescription, the label on the two medication vials and the appearance of the two medication vials are carefully reviewed with the patient [1].  It turns out that the pharmacy has made an incorrect delivery.  Apovit B- combin strong 250 pcs and Apovit B- combin strong 100 pcs have been given to the patient instead of Apovit B- combin strong 250 pcs and Apovit B1 vitamin strong 100 pcs.  The delivery error is explained to the patient. The patient is asked to go to the pharmacy with both the prescription and the supplied glasses and have the error corrected. It is explained that it is important that the patient receives the correctly prescribed preparations. |
| 24 | It stings at the urethra when I am urinating .  I have been to my own doctor and submitted a urine sample last week (30/08/2018), where they found nothing. The next day I called the doctor and wanted an appointment so the doctor could look at me. I didn't get an appointment, but the doctor said it was fungus and prescribed me Brentacort ointment. It doesn't say how long I should use it. It doesn't itch in the abdomen and my skin isn't irritated, so I don't think I have fungus myself. However, I have used the ointment anyway, without effect. It still stings when urinating.  What should I do? | TrygMedicin cannot diagnose the patient and, in this case , can only make a recommendation , and then refer on to their own doctor.  Immediately, it seems that there may have been a misunderstanding between doctor and patient in relation to symptoms and treatment.  Brentacort ointment for the treatment of burning during urination/fungus in the abdomen is questioned . For fungus, Brentan vaginal creame can be used.  The patient is recommended to contact a doctor and insist that the doctor looks at her before deciding on and choosing further treatment . The patient is also encouraged to submit a new urine sample to the doctor [1]. |
| 24 | I have just started treatment with Ciprofloxacin and I am now reading the package leaflet, which states a number of medicines that I must inform my doctor about if I am being treated with some of them. I am being treated with Theo-Dur, what should I do? | ​The patient is informed that we have been in contact with the prescribing doctor and that she has asked us to tell: the patient must take a break from Theo-Dur while she is being treated with ciprofloxacin .  A note is also written in SP. |
| 24 | Pt. calls TrygMedicin , after yesterday having been in ambulatorium . where she has received respectively Azithromycin and Trimbow added to his treatment for pneumonia and COPD respectively . Now she is worried about possible interaction with Azithromycin and Theodur .  And worried about possible effects on blood sugar in connection with the prescription of Trimbow (which was started yesterday ), Alnok and Furix (which she has, however, received over a longer period).  Pt is being treated with Metformin and measures blood sugar himself at home.  ​Interaction Azithromycin and Theodur ?  Influence of blood sugar on Trimbow , Alnok and Furix ? | It has been described that macrolides can increase the plasma concentration of theophylline modestly. The combination of Azithromycin and Theodur can, however, be used, as it is stated that it has no clinical significance for the treatment.  Regarding the effect on blood sugar, it is correct that Promedicin states that caution must be exercised when taking Furix in patients with latent or manifest diabetes mellitus.  However, the patient has been taking Furix for a long time and blood sugar should thus be regulated.  Nothing is stated about the effect on blood sugar in connection with the intake of tabl . Enough .  relating to. Trimbow states in ProMedicin that caution should be exercised with concurrent diabetes.  However, the patient measures blood sugar in advance and is encouraged to continue doing so and react if there are any irregularities. |
| 35 | During the interview, the patient asks about the following. If I feel like I have low blood sugar in the morning, should I take my rapid-acting insulin? | Rapid-acting insulin should not be used if blood sugar is too low. The patient is advised to eat sugary food if blood sugar is low. The patient is advised to measure his blood sugar before using fast-acting insulin if symptoms and doubts arise. |
| 46 | The questioner is in doubt as to when she should stop her treatment with or Klacid and Prednisone . Both treatments are started during hospitalization. She believes she must stop Klacid on 7/9-2018 and stop Prednisone on 12/9-2018.  The questioner has also received inhalation treatment during hospitalization but would like to know what it was the questioner received. The questioner experienced severe tremors from the treatment . Still have sporadic tremors which are subsiding, and wonder if it could still be from this treatment. | The questioner is confirmed that treatment with Prednisone must be stopped on 11/9-2018 and treatment with Klacid must be stopped on 7/9.  The questioner is informed that the questioner during hospitalization has been treated with Combivent , where tremor is a described side effect. The questioner is informed that it is unlikely that this treatment can still cause tremors and the questioner is advised to contact their own doctor if it does not disappear. |
| 43 | Patient asks during patient interview what Prednisolone does? | I inform the patient that Prednisolone is an adrenal cortical hormone which has an anti-inflammatory effect and therefore works against inflammation (1). |
| 75 | Can you take atorvastatin in the morning? | ​The atorvastatin dose can be taken at any time of the day. |
| 89 | Why do I get 2 x 300 mg gabapentin at night, but only one 300 mg tablet morning and evening? | On 15/06/18 the patient was started on fixed oxycodone 5 mg x 2 and gabapentin 300 mg x 2 for phantom pain (neuropathic pain) (1).  In the SP it is stated that the patient continues to have phantom pain on 21/06/18 (3).  The patient received an increased dose of gabapentin on 27/07/18 . The patient must have 300 mg in the morning and at noon and 600 mg at night. The change made by general practitioner (2).  Since the change in drug treatment was made by a general practitioner, I cannot read the rationale for the dose increase .  The recommended dosage for treatment with gabapentin in adults and children over 12 years of age is:   - 1st day 300 mg once daily. - 2nd day 300 mg 2 times daily. - 3rd day 300 mg 3 times daily.   After that, increase with 300 mg daily every 2-3 days. day to a maximum of 3,600 mg daily divided into 3 doses (4).  The patient's dose increase is probably due to the fact that the patient has not had sufficient effect to start the dose. In addition, it is normal for the dose to be increased gradually after the start of treatment. The advantage of having the largest dose at bedtime is that fatigue is avoided during the day and the patient may get an improvement in sleep quality. The patient is informed to contact his own doctor to get the full and correct explanation. |
| 89 | Can you become addicted to oxycodone ? | Oxycodone use can lead to physical and psychological dependence.  However, when the medicine is used according to the instructions for patients with chronic pain, the risk of developing physical and psychological dependence is significantly reduced |
| 93 | Are ondansetron and prednisolone drugs for nausea? | ​The patient is informed that ondansetron is a medicine for the prevention and treatment of nausea and vomiting caused by chemotherapy. In addition, the patient is informed that prednisolone is a drug that works against inflammatory conditions. |
| 108 | The patient says that during the hospitalization he got diagonosis ‘charcot foot’ and have pain here. During hospitalization he received good pain relief, but after discharge he has not been able to achieve satisfactory pain relief using paracetamol. He expresses a wish to be prescribed the same medication as on admission. | The patient is referred to their own doctor. |
| 108 | ​Patient says that he must be treated with clindamycin 300 mg capsule, 2 capsules morning, noon and evening for 10 days starting 08/11 and ending 18/11, but he has only been prescribed 20 tablets. Can it fit? | The patient needs 40 capsules of 300 mg clindamycin to complete his treatment. Therefore, 300 mg clindamycin capsules, 20 units, have been prescribed . x 2 packages. |
| 109 | ​Pt has had tablets with 665 mg paracetamol discontinued and now receives 500 mg paracetamol tablets instead .  Pt asks what the difference is between the two tablets and whether the effect is different. | ​The patient is informed that the medicinal substance in the two medicines is the same and therefore the effect is also the same.  The medicine with 665 mg of paracetamol is a depot formulation (1), thus paracetamol is released over a longer period of time and therefore works for a longer time. This can be advantageous if you have pain and need to sleep or if you need pain relief over a longer period and want to take fewer tablets. Tablets with 500 mg of paracetamol are advantageous to use if only single doses are to be taken, e.g. for headaches or if you need pain relief for a shorter period of time. |
| 109 | ​My home helper asked me to pick up my prolonged-release tablets from the pharmacy, but they said that Selozok and Kaleorid are the same. Shouldn't I take both pills? One box says something with Hexal .  Also, Unikalk is not on my medication list, should I continue with them?  I have also been given Lamictal 50 mg for pain, but only have to take half a tablet. But I can't share it with my fragile fingers, so shouldn't I just not take them? | ​Selozok and Kaleorid are two different medicines, so you must take one of each. But Selozok can be called something else, so that's probably what the pharmacy meant. Among other things, they can be called Metoprolol succinate , and they are white, while Kaleorid is brown.  If necessary, talk to the home nurse next time about the different tablets.  You must continue with your Unikalk unless your own doctor or the hospital's doctor says otherwise, so you must ask her.  Lamictal 50 mg cannot be split. You will need to call the department to get Lamictal 25 mg or arrange with them to stop. |
| 109 | I have previously received Panodil tablets 500 mg for my headache. Now the heart department has changed it to Panodil 665 mg and I don't think they work nearly as well as the others. My own doctor won't change it, so who should I contact? | The patient has been given a telephone number for the Heart Department. |
| 53 | ​I think lately I've been feeling a little "crazy": anxious, confused and had a strange uneasiness in my body. At the beginning of December, I started treatment with Mirtazapine at night and would therefore like to hear from you if my symptoms could be side effects to Mirtazapine ? | ​Yes, all the symptoms you mention are known side effects to mirtazapine and it is therefore not inconceivable that mirtazapine could be to blame for your symptoms. I would therefore recommend that you talk to your own doctor about the inconvenience of your treatment. |
| 138 | ​I have three questions about my medication after I was discharged on Monday (3 days ago):  I see that I have to take ( Cifin ) ciprofloxacin as a cure, but I haven't brought any pills home?  Simvastatin I am used to taking, but it is not on my list of medicines that I was given when I was discharged - should I take it?  I usually get 25 IU of insulin morning and evening, but the list says only 20 units morning and evening. What should I take? | Patient was admitted with col in exa and obs pneumonia [1].  ​Ciprofloxacin :  Patient was admitted with KOL in exa and was prescribed a course of prednisolone and ciprofloxacin . The course of ciprofloxacin was supposed to end on 1/15 and it seems that the patient should have dispensed 3 doses (1 on 1/14 and 2 on 1/15).  Solution: The patient has not finished his course. The patient wants to inform his own doctor about it, but does not want to do anything more.  Simvastatin :  During admission, there is a note stating that simvastatin is paused due to a slight suspicion of a side effect. At discharge, simvastatin has been discontinued.  Solution: Patient is happy to get rid of simvastatin and will inform his doctor about the change.  Insulin:  The patient is on permanent treatment with NovoMix and is used to taking 25 IU morning and evening. In FMK, the personal doctor has only noted "Dosage according to written agreement".  Just over a year ago, the patient was admitted to the geriatric ward, where the dose is set to 20 IU morning and evening. According to the patient, his own doctor subsequently changed the dose to 25 IU, but this cannot be seen from the prescription in FMK. At this admission, the dose from the last admission has been carried over (20 IU morning and evening).  Solution: Patient now knows that during hospitalization she only received 20 IU x 2 daily. She does not have the opportunity to see a doctor and have her blood sugar measured, but will speak to her own doctor tomorrow and agree whether she will continue with 20 or 25 IU. |
| 132 | The patient asks if she has been prescribed adrenal cortex hormone during her hospitalization. The patient has previously experienced side effects from adrenal cortex hormone and has experienced side effects similar to these after discharge. Side effects include dyspnoea, heavy legs and difficulty walking. The patient's experience of walking difficulties and heavy legs has gradually improved after discharge. | During hospitalization on 27/11/19, the patient was prescribed solu-medrol 80 mg iv x 1 and prednisolone 37.5 mg x 1 for 4 days (1).  Muscle weakness/ myopathy is a common side effect (1-10%) with solu-medrol treatment (2).  Muscle weakness/ myopathy is a very common side effect (> 10%) with prednisolone treatment (3).  The patient is informed that she has received adrenocortical hormone during her hospitalization and that these drugs can cause muscle weakness and thus an experience of heavy legs and difficulty walking. The patient's dyspnoea is probably due to heart and lung disease. |
| 140 | ​it is stated in the package insert that you should take flea seed shells at a different time in relation to some types of medicine. Does this also apply to diet?  Has been hospitalized with diarrhea and eats very little, and is therefore worried about whether sufficient nutrients are being absorbed from the diet. | There are no special restrictions or precautions in connection with food and the simultaneous intake of HUSK psyllium husks . |
| 155 | ​Patient (African woman) asks about the following:     - Is fish oil the same as vitamins? - Does Möllers Tran contain vitamins or only fish oil? - Picasol contain vitamins or only fish oil? - I take D vitamins. Is it ok to take multivitamins +50 ( Apovit ), Möller's Cod liver oil and D vitamins ( Apovit )? | The patient is recommended to consume only 1 tablet daily of multivitamins +50 ( Apovit ). If the patient wishes to consume fish oil at the same time, it is recommended to choose a fish oil without vitamins, e.g. Picasol .​ |
| 159 | ​Patient: I'm nervous about taking the Easyhaler ( Giona ) I got when I was discharged from hospital. I can see that you can have an allergic reaction by taking the inhaler and I have previously reacted violently with rashes and breathing difficulties to penicillin and another antibiotic. I therefore do not want to take the medicine. What should I do? | Easyhaler Giona contains budesonide and is an inhaled steroid.  There is no immediate cross-allergy between budesonide and penicillin [1].  Allergic reaction (including anaphylactic reaction and angioedema ) is known as a rare side effect (0.01-0.1%) [2].  This means that you do not have an increased risk of having an allergic reaction because you have reacted to penicillin, but there is a risk that you may react to Giona . The risk is very small, corresponding to 1 in 1000 - 10,000.  I think you should take the medicine and when you do it for the first time, make sure you have someone with you who can calm you down a bit. |
| 168 | Can you drink non-alcoholic beer, e.g. Nordic, when you are on antabuse treatment? | ​We cannot recommend that you consume alcohol-free beer during treatment with Antabuse. The limit for the content of alcohol in non-alcoholic beers and spirits is 0.5% by volume, corresponding to 1.3 g of pure alcohol, which is why there is still a small amount of alcohol in it.  An alternative for treating alcohol addiction is Campral. During treatment with this medicine, you do not experience a reaction when taking alcohol at the same time. |
| 168 | May I drive a car when I am being treated with Antabuse ( disulfiram )? | ​Antabuse ( disulfiram ) is not a drug which is marked with a red warning triangle (1). This does not affect the patient's ability to drive, and the patient is thus free to drive.  The patient is also advised to only drive if he considers it responsible. |
| 168 | Calling on behalf of his friend  and asks: how long does it take after Antabuse before you can drink a beer with your food? | ​Pt is advised to be completely abstinent himself - and peer management is encouraged to advise a friend to do the same. |
| 172 | ​Patient who has been admitted to L13 due to dyspnoea and cough and asks which inhalation medication he should take, as he has difficulty understanding what is stated in his discharge summary. Over time, he has been treated with up to six different inhalation preparations, which creates confusion. The patient can understand that he should not take Giona Easyhaler , Spiolto Respimat and Ventoline , but does not understand the reason and therefore asks why. | ​The patient is informed that he must take the following inhalation medication:     - Trimbow ( formoterol , glycopyrronium bromide and beclometasone ), 2 doses morning and evening (1).  *Formoterol is a long-acting β2-agonist (2).*  *Glycopyrronium bromide is a long-acting anticholinergic (2).*  *Beclomethasone is a glucocorticoid (2).* - Combivent ( ipratropium and salbutamol ), if necessary 1 dose (2.5 ml) no more than 2 times a day (1).  *Ipratripium is a short-acting anticholinergic (3).*  *Salbutamol is a short-acting β2-agonist (3).* - Bricanyl ( terbutaline ), if necessary 1 puff no more than 6 times a day (1).  *Terbutaline is a short-acting β2-agonist (4).*   The patient is informed that he should not take (1):   - Giona Easyhaler ( budesonide ).  *Budesonide is a glucocorticoid (5).* - Spiolto Respimat ( olodaterol and tiotropium ).  *Olodaterol is a long-acting β2-agonist (6).*  *Tiotropium is a long-acting anticholinergic (6).* - Ventolin ( salbutamol ).  *Salbutamol is a short-acting β2-agonist (7).*   Giona Easyhaler and Spiolto Respimat should not be taken as Trimbow contains a glucocorticoid equivalent to Giono Easyhaler and long-acting anticholinergic as well as long-acting sympathomimetic similar to Spiolto Respimat ​.    The reason why Ventoline should not be taken is that Combivent contains salbutamol and that Bricanyl has a similar effect.  The prescribing doctor has stated in the epicrisis that he wants to discontinue Combivent , but that the patient does not want this (1). In addition, the patient must complete the prednisolone course (1). |
| 193 | ​Patient has previously been treated with Methotraxat , where he experienced that it affected his mood. The patient needs to be treated with Methotrexate again and is concerned about the above. He asks if you can get depression from Methotrexate ? | Depression is an uncommon side effect of Methotrexate . Very common and common side effects of Methotrexate , such as decreased appetite, nausea and fatigue can affect mood. |
| 227 | The patient says that for several years she has been prescribed 2.5 mg Prednisolone daily. During his hospitalization and after discharge, the patient has had his dose increased to 37.5 mg daily for 1-2 weeks. On the same day that the patient decreased the dose, she became ill. The following days the patient gradually got better.  The patient would have liked to have asked about a week ago whether the Prednisolone should be downscaled , but since TrygMedicin had closed at the weekend it was not possible. For this reason, the question is now asked: should one downscale with Prednisolone ? | The patient is informed that it is necessary after longer treatment to gradually downscale, so that the body can adjust its own adrenal cortex production [1]. The doctor who made the patient's treatment plan has probably assessed that 1-2 weeks of treatment is not a long time and therefore it was not necessary to make a gradual downscaling. |
| 237 | ​The patient has been prescribed Flutiform inhalation spray 250+10 micrograms on 31/05/19 with the dosage of 2 doses in the morning and evening. He often experiences a limited effect of his inhalation medication at the end of the month. He asks if it could be because there is no more medicine in the inhalation device and if it is harmful to take an extra dose of a new inhalation spray. | ​The patient has used Flutiform for 27 days, corresponding to a total of 108 doses. Flutiform contains a total of 120 doses [1] and there should therefore be 12 doses left. However, it must be remembered that you often use a few doses before use to make sure that the device works. Therefore, the device can easily be empty. This can also be seen on the device , as a field turns red. We guide the patient to use a new device if he considers that the inhalation was not optimal. It is estimated that an "extra dose" of a new inhalation spray will not harm the patient. The patient says that he feels safe with this solution. |
| 237 | Male currently, 64 years old, lung function 20% experiencing isolated cases of malaise - is medicine the cause of this?  medicine :  Spiriva , 18 micrograms x 1 daily  Flutiform , dose x 2 daily  sep. 7/8719: Trimbow , dose = ? | It is currently being discussed with that it may be difficult to say whether the single incident is related to the patient's medication, but that he should be seen by his own doctor.  He seems to agree with this. |
| 237 | ​Last Friday I had my pneumovax . I have had different reactions afterwards and would like to know if it could be side effects to the vaccine - it is about soreness in the shoulder and shortness of breath (I already have COPD, but it has worsened)  On Monday I have to have the flu vaccine - is my reaction to pneumovax something that is not compatible with the fact that I already have to have my flu vaccine? - I am considering whether I should wait for the next vaccine. | Reaction at the injection site (tenderness) is a very common side effect of pneumovax .   Shortness of breath is not described as a side effect specifically. It is somewhat unknown whether some of the known side effects will result in shortness of breath.   The questioner states that he suffers from COPD and that he can feel fires being fired in wood-burning stoves in the area where he lives. From experience , it can affect his breathing, so we agree that he will look at it and contact his GP if it worsens.   Regarding influenza vaccine approx. 10 days after a pneumovax , then it's negotiable. In pro.medicin, the following stands for Pneumovax : Can be given at the same time as other vaccines, e.g. influenza vaccine, but in separate syringes and at different injection sites. |
