# Supplementary material for: Medicine information helpline after hospitalization–a randomized trial: Impact on patient satisfaction, patient concerns about medicines and clinical outcome on patient safety
Source: PLoS One. 2023 Oct 26;18(10):e0293523. doi: 10.1371/journal.pone.0293523 (PMC10602279; doi:10.1371/journal.pone.0293523)

**S7 Supporting Information:** Data showing means, medians and box plots for all data given in ordinal variables (five-point scale)

Group 1: Intervention group; Group 2: control group.

Question 1: Were you satisfied with the medicine information you received while being hospitalized?


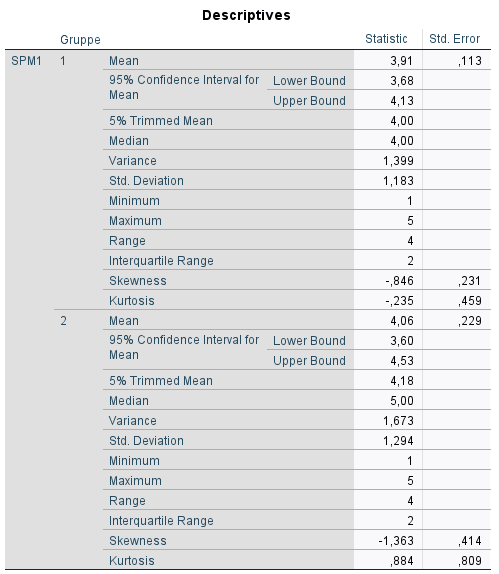


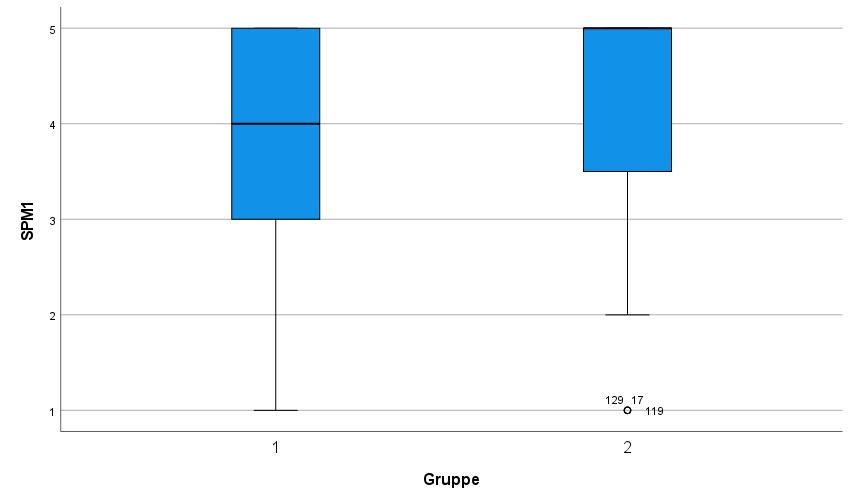


Question 4: Were you satisfied with the medicine information you received when you were discharged from the hospital?


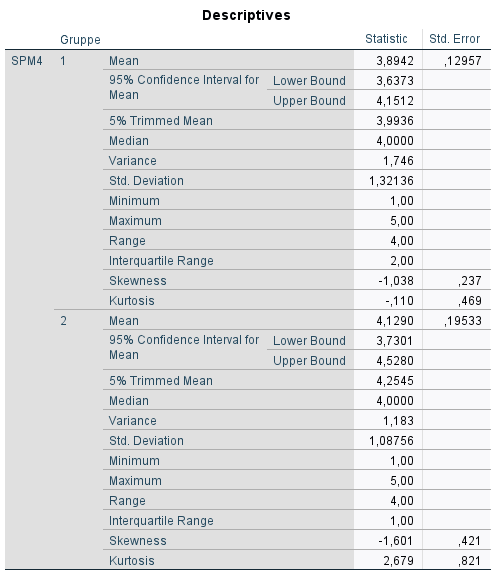


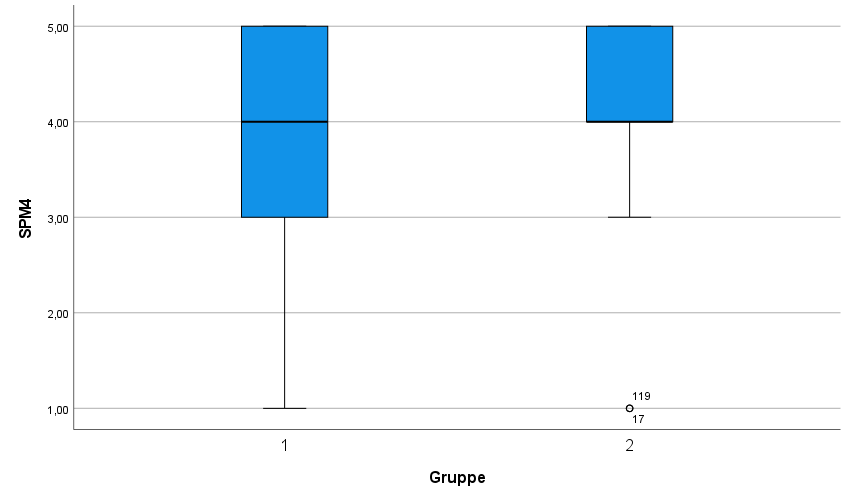


Question 9: Have you felt safe about your medication after you were discharged from the hospital?


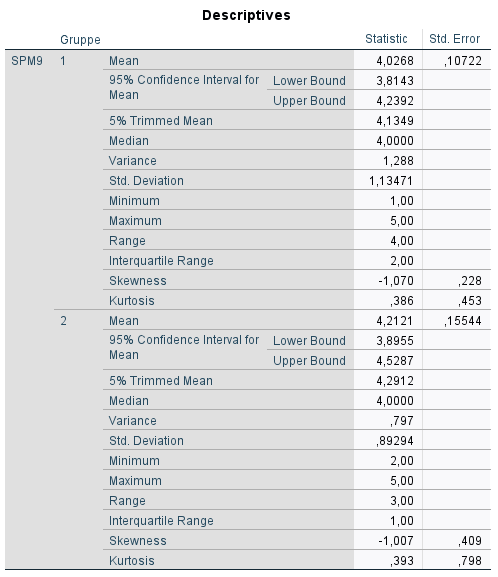


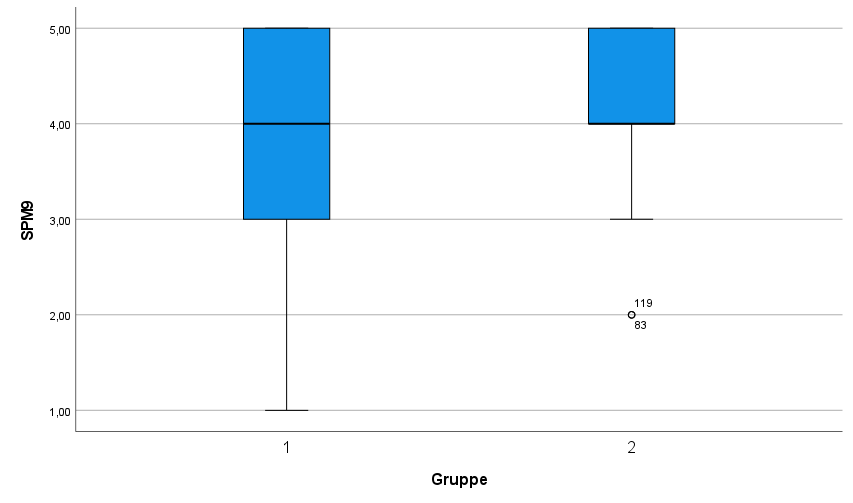


Question 10: My health. at present. depends on my medicines


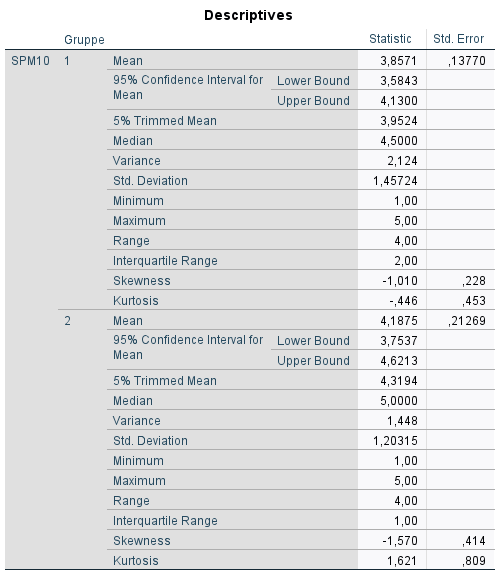


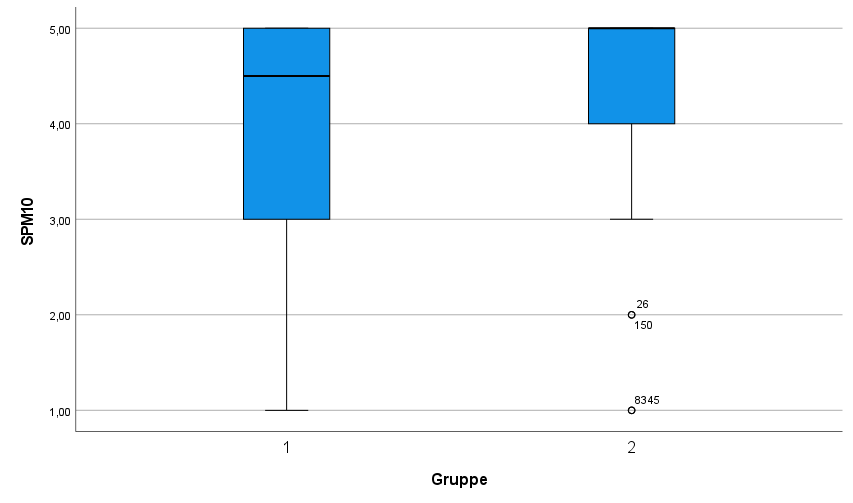


Question 11: Having to take medicines worries me


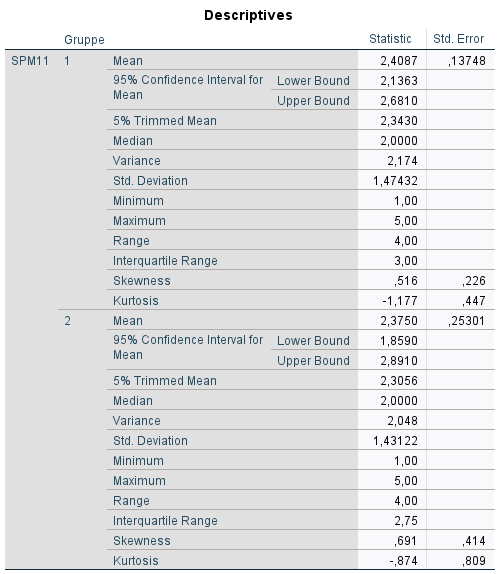


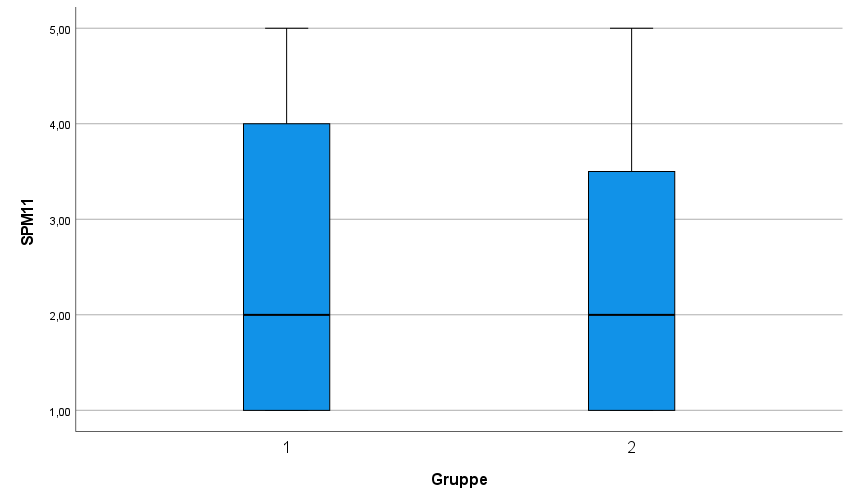


Question 12: My medicines are a mystery to me


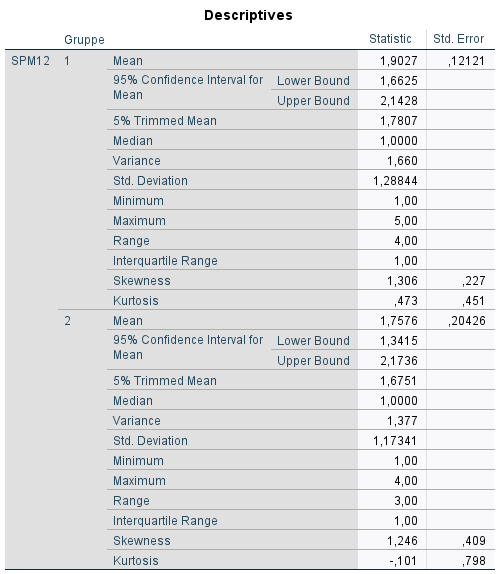


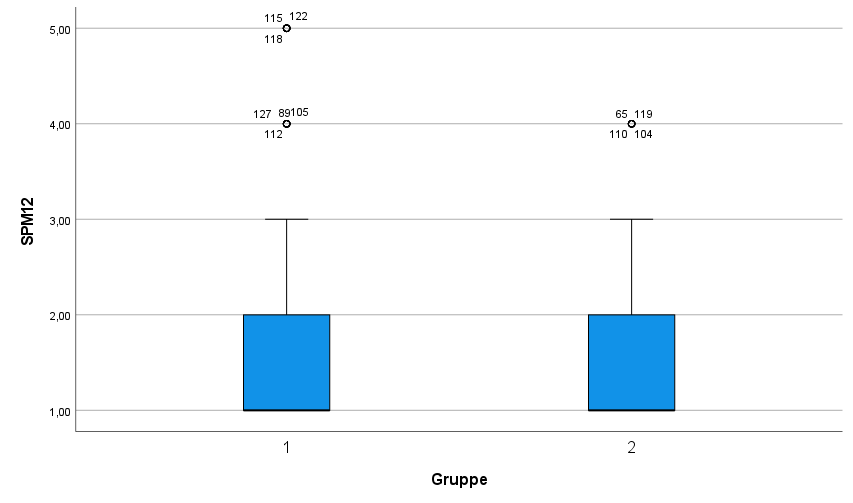


Question 13: My medicines disrupt my life


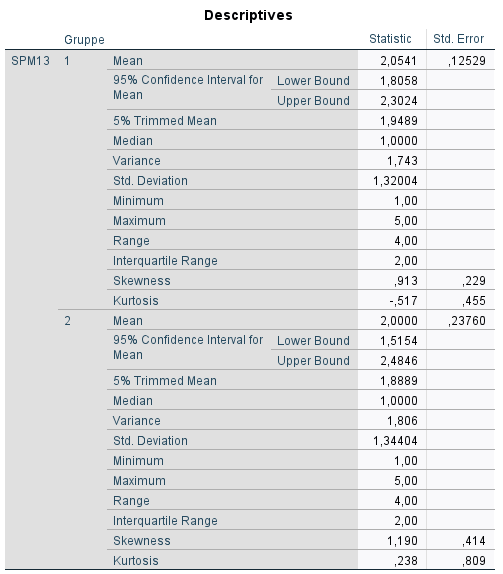


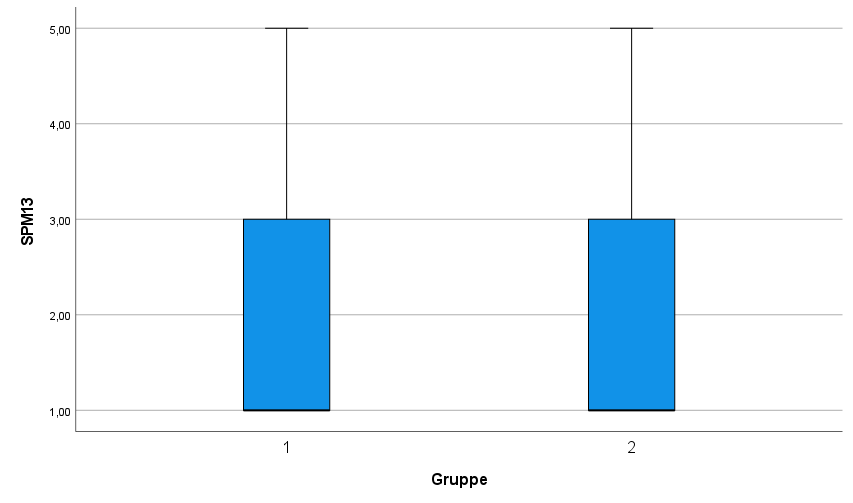


Question 19: Was the answer comprehensible?


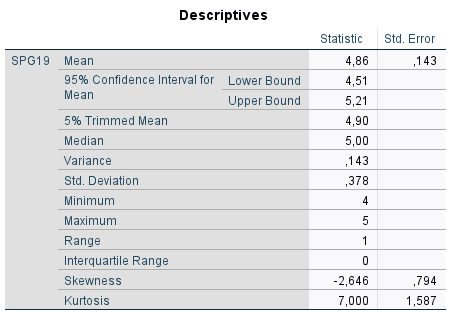


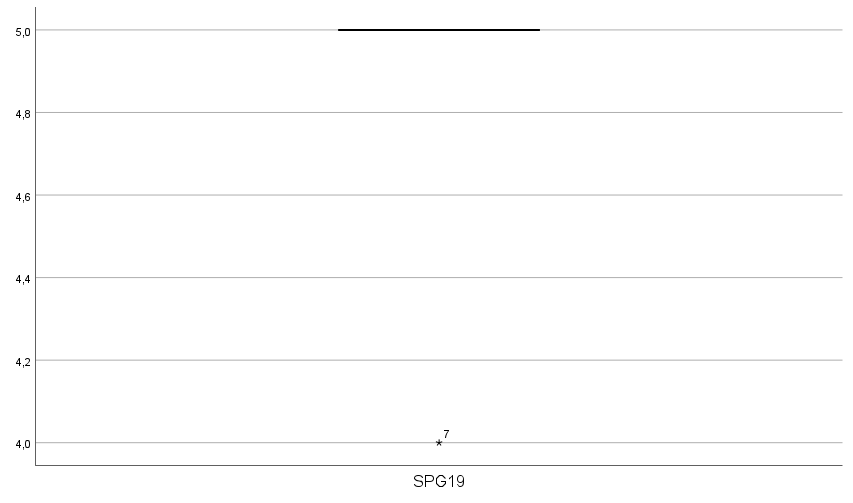


Question 20: Did you receive the information needed?


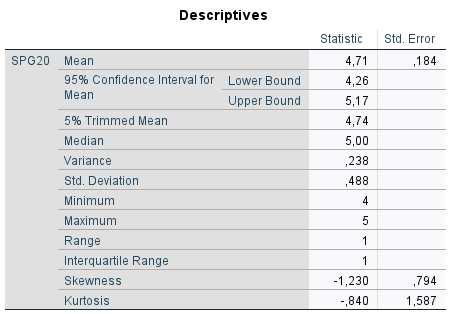


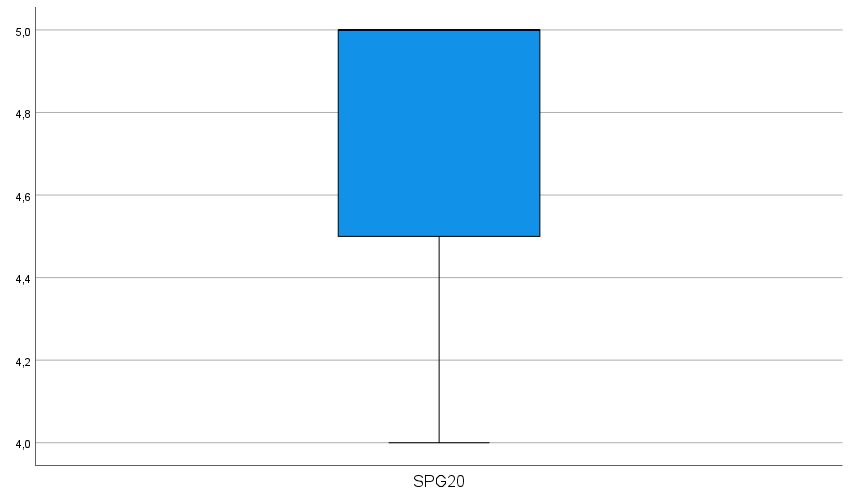


Question 21: Did the answer influence your medication consumption?


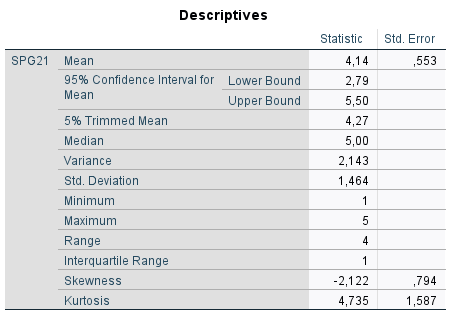


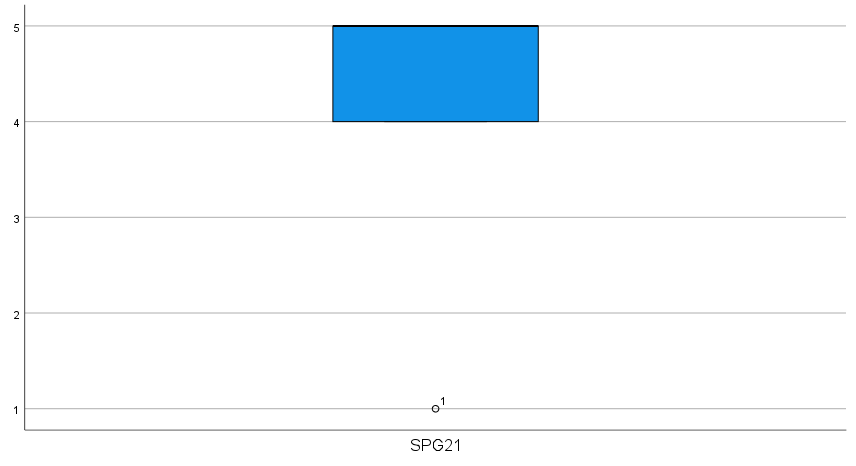


Question 22: Are you satisfied with the answer you received?


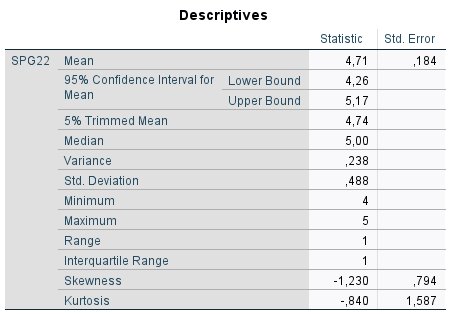


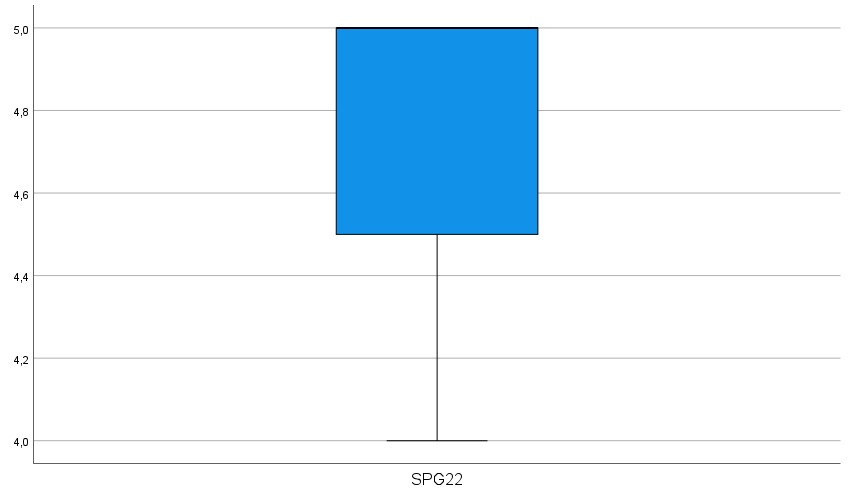


Question 23: Have you felt safe regarding your medication after you contacted the MIH?


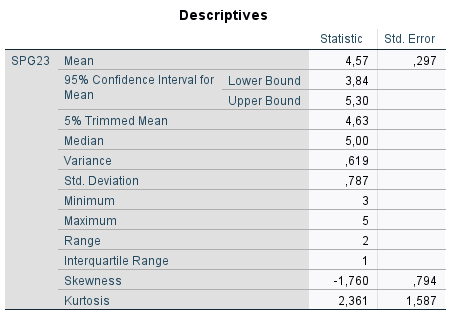


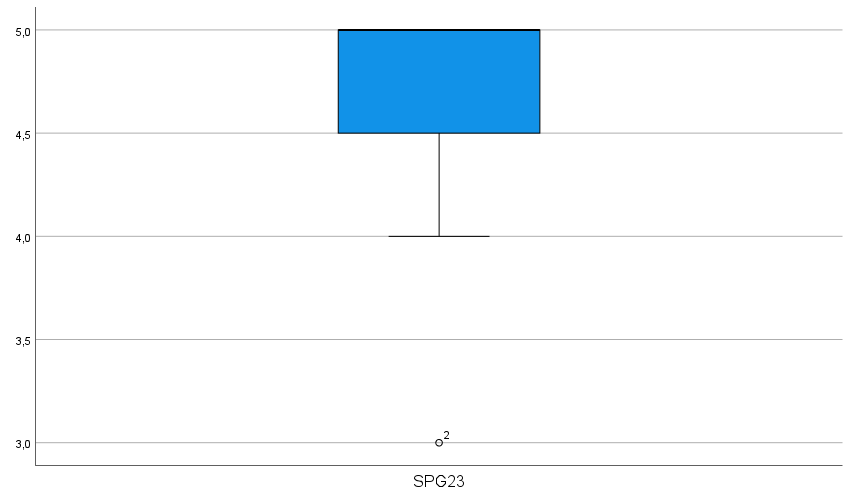


Question 25: Are you satisfied with the MIH?


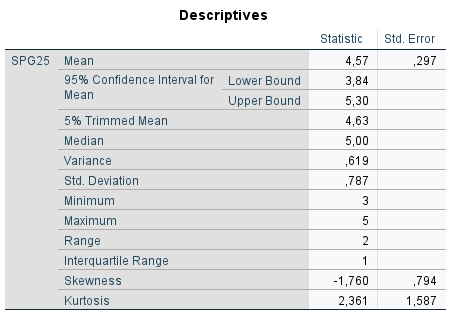


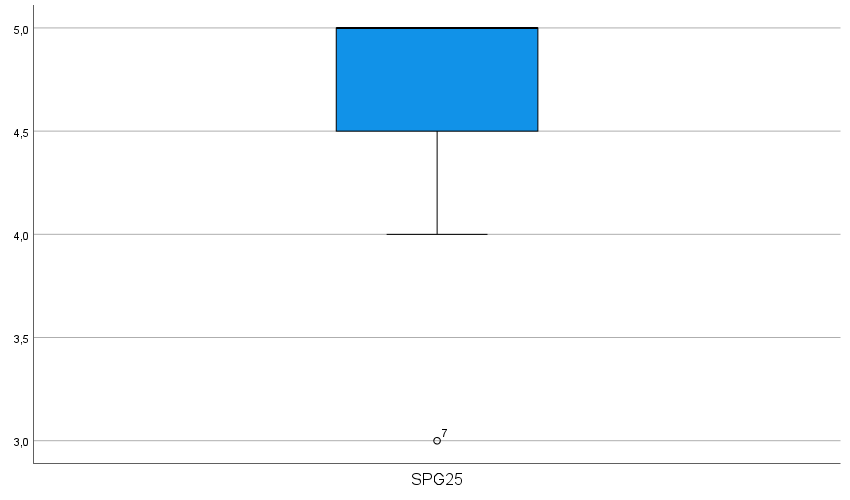

Supplement: S4 File — (DOCX) [file pone.0293523.s007.docx]
