# Supplementary material for: Medicine information helpline after hospitalization–a randomized trial: Impact on patient satisfaction, patient concerns about medicines and clinical outcome on patient safety
Source: PLoS One. 2023 Oct 26;18(10):e0293523. doi: 10.1371/journal.pone.0293523 (PMC10602279; doi:10.1371/journal.pone.0293523)
Supplement: Attachment — Submitted filename: PONE-D-22-33407_reviewer.pdf [file pone.0293523.s008.pdf]

PLOS ONE

Medicine Information Helpline after hospitalization – a randomized trial: impact on patient satisfaction, patient belief in medicine and clinical outcome on patient safety

--Manuscript Draft--

**Manuscript Number:**	PONE-D-22-33407
**Article Type:**	Clinical Trial
**Full Title:**	Medicine Information Helpline after hospitalization – a randomized trial: impact on patient satisfaction, patient belief in medicine and clinical outcome on patient safety
**Short Title:**	Medicines information to discharged patients
**Corresponding Author:**	Marianne Hald Clemmensen, Ph.D.<br>Danish Medicines Agency<br>Virum, DENMARK
**Keywords:**	Hospital, hospital discharge, medication changes, medication related problems, Medicine Information Helpline, patient safety, drug safety, patient satisfaction, patient information, Patient beliefs, Denmark, clinical trial
**Abstract:**	Background and aim: Hospitalization often leads to changes in patients' medicine which challenges a safe medication after discharge. Medicine information helplines (MIHs) can be valuable for patients in overcoming these challenges.<br>This study evaluates patient satisfaction with a new established Danish hospital-based MIH for discharged patients by exploring how the service affects the patient's belief and perception of security about their medical treatment, followed by an assessment of the clinical impact of MIH on patient safety.<br>Method: A randomized controlled study design was used in the present study. The study was registered at clinicaltrials.gov with the identification number NCT03829995. Participants were randomization 1:4 (50:200) into a control- and intervention group. Participants in the control group were offered standard care and those in the intervention group were offered access to the MIH. A telephone interview performed 2-4 weeks after discharge assessed patient satisfaction with the helpline, patient belief about medicine and the feeling of security in relation to medicine. Medication-related problems (MRPs) were identified and clinical impact of the MIH service was assessed.<br>Results: A total of 250 participants were included in the study and 152 participated in the interviews (33 control and 119 intervention). Thirty-seven questions were enquired by 26 participants to the MIH during the study, and they all expressed a high satisfaction with the MIH. Participants in the intervention group reported that the MIH increased their sense of security regarding medicine. Assessment of the enquiries revealed at least one MRP per enquiry, and in most cases the advice given had a high- or moderate clinical significance.<br>Conclusion: The MIH was highly appreciated by the participants, which indicate that the MIH is a valuable service for discharged patients in improving the sense of security in relation to medication and alleviating MRPs. Providing easy access for patients to medicine information may indeed improve a patient safe medicine use after discharge.
**Order of Authors:**	Karianne Wilhelmsen Fjære, MSc<br>Tim Emil Vejborg, MSc<br>Lene Colberg, MSc<br>Cecilia Strøjer Ulrich, MSc<br>Lars Pedersen, MD, PhD<br>Ann Kathrin Demény, MD<br>Joo Hanne Poulsen, Ph.D.<br>Helle Byg Armandi, MSc<br>Marianne Hald Clemmensen, Ph.D.
**Additional Information:**	

Powered by Editorial Manager® and ProduXion Manager® from Aries Systems Corporation

Question	Response
**Financial Disclosure**<br><br>Enter a financial disclosure statement that describes the sources of funding for the work included in this submission. Review the submission guidelines for detailed requirements. View published research articles from *PLOS ONE* for specific examples.<br><br>This statement is required for submission and **will appear in the published article** if the submission is accepted. Please make sure it is accurate.<br><br>**Unfunded studies**<br>Enter: *The author(s) received no specific funding for this work.*<br><br>**Funded studies**<br>Enter a statement with the following details:<br>• Initials of the authors who received each award<br>• Grant numbers awarded to each author<br>• The full name of each funder<br>• URL of each funder website<br>• Did the sponsors or funders play any role in the study design, data collection and analysis, decision to publish, or preparation of the manuscript?<br>• **NO** - Include this sentence at the end of your statement: *The funders had no role in study design, data collection and analysis, decision to publish, or preparation of the manuscript.*<br>• **YES** - Specify the role(s) played.<br><br>* typeset	MHCL received one grant supporting the research presented in the article.<br>No other authors received any grants or awards.<br>The grant was received from The Danish Research Unit for Hospital Pharmacy, Amgros I/S, Copenhagen, Denmark.<br>https://amgros.dk/om-amgros/samarbejdspartnere/nationalt-samarbejde/<br>The funders did not play any role in study design, data collection and analysis, preparation of the manuscript or in the decision to publish the data. The grant only finansed a small part of the total costs of the study.
**Competing Interests**<br><br>Use the instructions below to enter a competing interest statement for this submission. On behalf of all authors, disclose any competing interests that could be perceived to bias this work—acknowledging all financial support and any other relevant financial or non-financial competing interests.	The authors have declared that no competing interests exist.

This statement is required for submission and **will appear in the published article** if the submission is accepted. Please make sure it is accurate and that any funding sources listed in your Funding Information later in the submission form are also declared in your Financial Disclosure statement.<br><br>View published research articles from *PLOS ONE* for specific examples.<br><br>**NO authors have competing interests**<br><br>Enter: *The authors have declared that no competing interests exist.*<br><br>**Authors with competing interests**<br><br>Enter competing interest details beginning with this statement:<br><br>*I have read the journal's policy and the authors of this manuscript have the following competing interests: [insert competing interests here]*<br><br>* typeset	
**Ethics Statement**<br><br>Enter an ethics statement for this submission. This statement is required if the study involved:<br><br>• Human participants<br>• Human specimens or tissue<br>• Vertebrate animals or cephalopods<br>• Vertebrate embryos or tissues<br>• Field research<br><br>Write "N/A" if the submission does not require an ethics statement.<br><br>General guidance is provided below. Consult the submission guidelines for detailed instructions. **Make sure that all information entered here is included in the Methods section of the manuscript.**	The protocol was approved by the Danish Data Protection Agency (Jnl No. VD-2018-214, I-Suite No. 6459). The National Committee on Health Research Ethics assessed that the study did not require an ethical approval according to Danish law (Jnl No. H-18003889). Participants were informed both verbally and in writing about the nature of the study, the publication plans and that they could withdraw from the study at any time without further consequences. All participants signed a consent form and were anonymized in the study. The study is registered at clinicaltrials.gov with the identification number NCT03829995.

**Format for specific study types**<br><br>**Human Subject Research (involving human participants and/or tissue)**<br>• Give the name of the institutional review board or ethics committee that approved the study<br>• Include the approval number and/or a statement indicating approval of this research<br>• Indicate the form of consent obtained (written/oral) or the reason that consent was not obtained (e.g. the data were analyzed anonymously)<br><br>**Animal Research (involving vertebrate animals, embryos or tissues)**<br>• Provide the name of the Institutional Animal Care and Use Committee (IACUC) or other relevant ethics board that reviewed the study protocol, and indicate whether they approved this research or granted a formal waiver of ethical approval<br>• Include an approval number if one was obtained<br>• If the study involved *non-human primates*, add *additional details* about animal welfare and steps taken to ameliorate suffering<br>• If anesthesia, euthanasia, or any kind of animal sacrifice is part of the study, include briefly which substances and/or methods were applied<br><br>**Field Research**<br><br>Include the following details if this study involves the collection of plant, animal, or other materials from a natural setting:<br>• Field permit number<br>• Name of the institution or relevant body that granted permission	
**Data Availability**<br><br>Authors are required to make all data underlying the findings described fully available, without restriction, and from the time of publication. PLOS allows rare exceptions to address legal and ethical concerns. See the PLOS Data Policy and FAQ for detailed information.	No - some restrictions will apply

A Data Availability Statement describing where the data can be found is required at submission. Your answers to this question constitute the Data Availability Statement and **will be published in the article**, if accepted.<br><br>**Important:** Stating 'data available on request from the author' is not sufficient. If your data are only available upon request, select 'No' for the first question and explain your exceptional situation in the text box.<br><br>Do the authors confirm that all data underlying the findings described in their manuscript are fully available without restriction?	
**Describe where the data may be found in full sentences. If you are copying our sample text, replace any instances of XXX with the appropriate details.**<br><br>• If the data are **held or will be held in a public repository**, include URLs, accession numbers or DOIs. If this information will only be available after acceptance, indicate this by ticking the box below. For example: *All XXX files are available from the XXX database (accession number(s) XXX, XXX.*).<br>• If the data are all contained **within the manuscript and/or Supporting Information files**, enter the following: *All relevant data are within the manuscript and its Supporting Information files.*<br>• If neither of these applies but you are able to provide **details of access elsewhere**, with or without limitations, please do so. For example:<br><br>*Data cannot be shared publicly because of [XXX]. Data are available from the XXX Institutional Data Access / Ethics Committee (contact via XXX) for researchers who meet the criteria for access to confidential data.*<br><br>*The data underlying the results presented in the study are available*	Some data cannot be shared publicly because of patient specific information. The data are available in an anonymised form from the The Hospital Pharmacy, Capital Region of Denmark (contact via medicininfo@regionh.dk) for researchers who meet the criteria for access to confidential data.

from (include the name of the third party and contact information or URL).<br>• This text is appropriate if the data are owned by a third party and authors do not have permission to share the data.<br><br>* typeset	
Additional data availability information:	

Powered by Editorial Manager® and ProduXion Manager® from Aries Systems Corporation

Manuscript
Click here to access/download;Manuscript;Paper_2023 01 11.docx

Medicine Information Helpline after hospitalization – a randomized trial: impact on patient satisfaction, patient belief in medicine and clinical outcome on patient safety

Short title:
Medicines information to discharged patients

Karianne Wilhelmsen Fjære[1¶]; Tim Emil Vejborg [1¶]; Lene Colberg[1¶]; Cecilia Strøjer Ulrich[1&]; Lars Pedersen[2&]; Ann Kathrin Demény[3&]; Joo Hanne Poulsen[1&]; Helle Byg Armandi[1&]; Marianne Hald Clemmensen[1¶*]

1: Medicines Information Center, The Hospital Pharmacy, Capital Region of Denmark, Bispebjerg Hospital, Esther Ammundsens Vej 24B DK-2400 Copenhagen NV, Denmark

2: Department of Respiratory Medicine, Capital Region of Denmark, Bispebjerg Hospital, Ebba Lunds Vej 44 DK-2400 Copenhagen NV, Denmark

3: Emergency Department, Capital Region of Denmark, Bispbjerg Hospital, Ebba Lunds Vej 44 DK-2400 Copenhagen NV, Denmark

*Corresponding author
E-mail: haldlarsen@hotmail.com (MHC)

[¶]These authors contributed equally to this work.

[&]These authors also contributed equally to this work.

The study is registered at clinicaltrials.gov with the identification number NCT03829995.

Abstract

Background and aim: Hospitalization often leads to changes in patients' medicine which challenges a safe medication after discharge. Medicine information helplines (MIHs) can be valuable for patients in overcoming these challenges.

This study evaluates patient satisfaction with a new established Danish hospital-based MIH for discharged patients by exploring how the service affects the patient's belief and perception of security about their medical treatment, followed by an assessment of the clinical impact of MIH on patient safety.

Method: A randomized controlled study design was used in the present study. The study was registered at clinicaltrials.gov with the identification number NCT03829995. Participants were randomization 1:4 (50:200) into a control- and intervention group. Participants in the control group were offered standard care and those in the intervention group were offered access to the MIH. A telephone interview performed 2-4 weeks after discharge assessed patient satisfaction with the helpline, patient belief about medicine and the feeling of security in relation to medicine.

Medication-related problems (MRPs) were identified and clinical impact of the MIH service was assessed.

Results: A total of 250 participants were included in the study and 152 participated in the interviews (33 control and 119 intervention). Thirty-seven questions were enquired by 26 participants to the MIH during the study, and they all expressed a high satisfaction with the MIH. Participants in the intervention group reported that the MIH increased their sense of security regarding medicine.

Assessment of the enquiries revealed at least one MRP per enquiry, and in most cases the advice given had a high- or moderate clinical significance.

Conclusion: The MIH was highly appreciated by the participants, which indicate that the MIH is a valuable service for discharged patients in improving the sense of security in relation to medication and alleviating MRPs. Providing easy access for patients to medicine information may indeed improve a patient safe medicine use after discharge.

Introduction

Healthcare systems are becoming more and more complex and patients must navigate between hospitals and the primary sector. Transition from hospitals to private homes is known to challenge the patient safety. Recent studies highlight that adverse events related to cross-sectorial care are increasing of which many can be attributed to errors in the medication process [1-3]. During hospitalization patients often experience changes in their medication, which can lead to confusion and insecurity about the new or changed medicine after discharge [1, 3]. Further, patients report that they did not receive important information about their medication upon hospital discharge [2, 4, 5]. It is estimated that up to 65 % of patients discharged from a hospital subsequently experience medication-related problems (MRPs) [3] .

Importantly, it has been shown that patient education and counseling can reduce the risk of medicine related adverse events and rehospitalization, and patients have been identified as a key resource in improving medicines safety in cross-sector situations [1, 2, 4]. Thus, there is a need to ensure that patients receive the relevant support, information and education related to their medicine.

Medicine Information Helplines (MIH) can play an important role in supporting patients in a safe medication after hospitalization. In the UK, MIH services were established in the early 1990s enabling patients discharged from the hospital direct access to communication with a pharmacy professional to improve the medication safety and to reduce medication errors [6]. MIH could also support a better medication adherence by empowering the patients through provision of knowledge and information about their use of medicines [1, 7-10]. Further, patients are generally satisfied with the MIH and the information received about their medicines [1, 5, 7, 8, 11, 12].

Up until 2018, discharged patients in Denmark have not had access to a MIH. Patient may enquire the general practitioners or community pharmacies with medicine-related questions [9]. However,

this is not always suitable, as the new medicine may be used in hospitals only, the general practitioners may be unavailable, or the community pharmacy does not have access to the discharge summary from the hospital. Therefore, the Danish hospital-based service, MIH, was established in 2018 for discharged patients in the Capital Region of Denmark [13]. This study is to our knowledge the first to explore if addition of MIH affects patient's beliefs and perception of security about medicines as compared to standard care with limited medicine information support after discharge. Furthermore, the aim was to evaluate patient satisfaction with the new MIH followed by an assessment of the clinical impact of MIH on patient safety.

Method

Setting

The MIH was operated by experienced pharmacists and a pharmacy technician from the Medicine Information Centre (MIC) at the Hospital Pharmacy at Bispebjerg Hospital in the Capital Region of Denmark.

An acute care setting and a non-acute inpatient setting were purposively chosen to obtain possible variations in the clinical impact related to the MIH, and the impact on patient satisfaction, belief, and perception of security in medicine after discharge. Thus, the Emergency Department and the Department of Respiratory Medicine were selected.

Recruitment of participants

A total of 250 patients were recruited in the study from July 2018 through May 2019. The patients were recruited in the hospital wards by the first three authors from MIC. All admitted patients at the two wards were assessed and eligible participants were subsequently approached for enrollment. Eligible participants were 18 years or older, spoke and understood Danish, and were scheduled for discharge to their private homes within a few days after enrollment. Exclusion criteria were related to severe dementia diagnosis, terminally illness, isolation precautions, aphasia, suicidal or patients in custody.

Participation in the study was voluntarily and all were given oral and written information about the study, publication plans and his or her right to withdraw from the study at any time without further consequences. All participants signed a consent form and patients' participation was documented in their hospital files by the clinical pharmacists.

Study design

A randomized controlled study design was used to explore the study objective.

The included participants were randomized 1:4 (50:200) into a control and an intervention group with parallel assignment using the randomization online system randomization.com. The randomization was blinded and divided into block sizes of 5/10/15/20/25 with sequent numbered, opaque sealed envelopes.

The control group (50 participants) received no intervention beyond standard care which does not include access to any information services post discharge. The intervention group (200 participants) were offered standard care and the opportunity to contact the MIH on all business days from 8 am to 3.30 pm, should any medicine-related questions arise after hospital discharge.

Upon enrolment patients in the intervention group received a card with contact information for the MIH. All enquiries to the MIH were answered orally and/or written by either a pharmacist or a pharmacy technician from the MIC. Potential questions were documented in the MIC's database and categorized into predefined types of categories, such as adverse effects, choice of therapy, dosage/administration, interactions and product information.

Telephone interviews

A primary outcome of the present study was to evaluate patient's beliefs and perception of security about medicines as compared to standard care. Further, patient satisfaction with the new MIH was included as a primary outcome. Both primary outcomes were investigated through telephone interviews with the included participants

An interview guide was developed to assess patient satisfaction with the medicine information obtained during hospitalization, at discharge and by the MIH. In order to investigate patient's beliefs and perception of security about medicines already validated questions from the Beliefs About Medicine Questionnaire proposed by Horne et al. (1999) [14] and from a study by Badiani et al. (2017) [7] were included in the interview guide. Further questions were included according to the study objectives and contextual settin[illegible]he guide was divided into three overall sections directed towards the participants in the control and intervention group. The interview guide was semi-structured and contained both closed- and open-ended questions or statements, from where the participants would rate the statements using a five-point scale (1=not at all and 5=to a very great extent), see S2 Appendix. However, if a patient is not in any medical treatment prior to, during admission or after discharge, the patient is not able to provide an answer to each question from the interview guide. Hence, the total number of respondents to each question may vary. The questions were pilot tested by 12 patients and 5 healthcare professionals, and the guide was subsequently optimized accordingl[illegible]ll eligible participants were interviewed by telephone within 2-4 weeks after discharge.

Participants were rated as non-responders if they declined to be interviewed or if they did not answer the phone within five attempts on five different days over a two week perio[illegible] addition, participants were also rated as a non-responder if they showed cognitive or/and communicative problems during the interview, making it difficult to complete the interview, or if the patient was readmitted or deceased[illegible]

Clinical impact assessment

A secondary outcome of the present study was to assess the clinical impact of MIH on patient safety. To do this, all enquiries were analyzed and categorized for MRPs and pharmacy interventions according to the Westerlund System, see table 1 [15, 16]. Furthermore, the impact on patient safety was assessed using the DOCUMENT system where the clinical significance of each enquiry from the intervention group was determined individually by two clinical pharmacists (the

first and fourth author[illegible]7]. Any discrepancies were discussed in order to determine a concluding assessment of the clinical significance, see table 1.

Table 1 – Clinical impact assessment of MRPs and the pharmacy intervention.

**MRPs classified using the Westerlund System [15, 16]**
Uncertainty about aim of the medicine; adverse reaction; interaction; therapy failure; problem of administration; inappropriate time for the medication intake/wrong dosage interval; over-/underuse of the medicine, medicine duplication; inappropriate storage of the medicine; difficulty opening the medicine; other MRPs.
**Clinical significance of the pharmacy intervention described by the DOCUMENT system [17]**
**Nil:** No consequence to the patient
**Low:** Consequences to the patient are related to costs or information only
**Mild:** Consequences to the patient are that they have improved compliance or improved or prevented a minor symptom. The sign or symptom should not require a doctor's visit to treat
**Moderate:** When, if the intervention did not occur, it was likely that the patient would have had to visit the doctor because of the consequences. Also covers the situation where the pharmacist needs to refer the patient to the doctor because of the seriousness of the situation
**High:** When, if the intervention did not occur, it was likely that the patient would have<br>- had to go to a hospital because of the consequences. Also covers the situation where the pharmacist needs to refer the patient to a hospital because of the seriousness of the situation<br>- required assistance from a regular nurse visit, or would have required placement into residential care

Data analysis

No similar studies are available from which a sample size can be calculated.

This study randomized controlled study design, and it was set to detect a 10 % improvement on a 5-point scale measuring patient satisfaction with medicine information and patient concerns and beliefs about medicine. From the literature, a mean score of 3.5 ± 0.5 is anticipated [2, 11, 18, 19]. The 10% was set by the authors as this was assessed to allow for detection of any relevant changes in the scores.

Power was set to 90 % with a statistical significance level of 0.05. Thus, a sample size of 43 patients was calculated, but to adjust for dropouts, the sample size was set to 50 patients. It was estimated by the authors that 20% of the patients in the intervention group would contact the MIH thus resulting in a (50:200) (control:intervention) allocation.

Quantitative data from the patient interviews were analyzed in Microsoft Excel (for Office 365 MSO) and IBM SPSS (Version 25) with descriptive statistics and simple parametric statistics. Mann-Whitney U test were used for analysis of ordinal variables (five-point scale) and Chi-Square test were used for nominal variables (gender, yes/no). An independent t-test was used to test for difference in age and number of medications by admission and discharge.

Ethics approval

The protocol was approved by the Danish Data Protection Agency (Jnl No. VD-2018-214, I-Suite No. 6459). The National Committee on Health Research Ethics assessed that the study did not require an ethical approval according to Danish law (Jnl No. H-18003889). Due to academic considerations one of the outcome measures (quality of life vs. beliefs about medicines) was changed before the study was commenced. However, this amendment did not affect the overall methodology or the type of data collected and thus no further approval form the Ethics Committee was necessary. Participants were informed both verbally and in writing about the nature of the study, the publication plans and that they could withdraw from the study at any time without further consequences. All participants signed a consent form and were anonymized in the study. The authors confirm that all ongoing and related trials for this drug/intervention are registered at the clinicaltrials.gov with the identification number NCT03829995

Results

Participants

A total of 946 patients were assessed for eligibility at the two hospital wards. Of these, 319 patients failed to meet the inclusion criteria and 377 patients either declined the invitation to participate, were asleep, discharged or transferred to another ward at the time of inclusion.

Thus, 250 participants were included in the study and randomized into a control group (n=50) and an intervention group (n=200). In the follow-up period, 98 participants (17 in the control group and 81 in the intervention group) were considered as non-responders. Finally, 33 participants were interviewed in the control group and 119 participants the intervention group, see Figure 1.

Figure 1: Consort study diagram showing the flow of invited, included, excluded and dropout patients.

The participants were included equally from the Emergency Department and from the Department of Respiratory Medicine, and their characteristics are presented in table 2. No significant differences were found between control and intervention group regarding gender, age or number of medications at admission or discharge respectively, see table 2.

Table 2 - Characteristics of enrolled participants.

Characteristics	Control group (n = 33)	Intervention group (n = 119)	All participants (n=152)	P Value
Sex, No. (%)[a]				
Male	13 (39)	55 (46)	68 (45)	0.540
Female	20 (61)	64 (54)	84 (55)	0.540
Age, median (IQR)	71 (57-78)	67 (51-74)	68 (54-75.5)	0.143
Hospital ward, No. (%)[a]				
Acute medical ward	12 (36)	60 (50)	72 (47)	0.152
Department of respiratory medicine	21 (64)	59 (50)	80 (53)	0.152
No. of medications by admission, median (IQR)	7 (5-12.5)	8 (4-13)	7,5 (4-12.5)	0.624
No. of medications by discharge, median (IQR[illegible]	9 (5.5-14.5)	9 (5-14)	9 (5-14)	0.724

IQR: interquartile range. [a]Percentages of total number.

Enquiries to the MIH

During the study period, the participants from the intervention group were offered the possibility to contact the MIH if they had any questions regarding their medication after discharge.

In all, 26 participants raised a total of 37 enquiries to the MI[illegible]nd the enquiries were categorized into predefined types of categories. The majority of enquiries were related to dosage/administration and product information (effect and usage), see table 3.

Table 3 – Categorization of enquiries to MIH from July 2018 through May 2019.

**Category**	Enquiries **before** interview	Enquiries **during** interview	Enquiries **after** interview
Adverse effects, No. (%)	1 (2.7)	2 (5.4)	3 (8.1)
Choice of therapy, No. (%)	1 (2.7)	0 (0.0)	1 (2.7)
Dosage & administration, No. (%)	3 (8.1)	5 (13.5)	3 (8.1)
Interactions, No. (%)	1 (2.7)	0 (0.0)	3 (8.1)
Product information, effect and usage, No. (%)	4 (10.8)	6 (16.3)	1 (2.7)
Other, No. (%)	0 (0.0)	1 (2.7)	2 (5.4)
**Total number**	**10 (27%)**	**14 (38%)**	**13 (35%)**

"Other" include enquiries about patients' control/treatment at the hospital and the need to speak with either a hospital- or general physician.

Medicine information during hospitalization and after discharge

A total of 152 participants were interviewed by telephone and the interview guide in S2 Appendix was followed. For nominal data, participants answering either "yes", "no", "do not know" were included in the analysis, whereas ordinal data only included answers containing ratings from 1-5. Not all participants answered all questions. Thus, in the following, the number of answers given to each specific question is represented by "n", and the results are provided with the calculated percentage based on the fraction of "n".

In the control group 55% of the participants (18/33) reportedly experienced a chang[illegible] their medication during hospitalization, see S3 appendix. A similar level of change in medication was reported by participants in the intervention group (58%, 68/118), see S3 appendix. Further, the participants expressed a high leve[illegible] satisfaction with the medicine information provided during

hospitalization and at discharge (figure 2). No significant differences were seen between the control- and intervention group.

Figure 2 – Patient satisfaction with medicine information provided during hospitalization and after discharge.

Patients scored satisfaction on a 5-point scale with 1=not at all and 5=to a very great extent. No significance was found between control- and intervention group (Mann-Whitney U test, $p>0.05$).

Approximately half of the participants (48 %, 73/141) preferred receiving their medicine information both written and orally, and 2/3 of these expressed that this combination gave them the opportunity to validate and repeat the obtained information when settled at home. Of the participants in the control and intervention group, 21% (7/33) and 29% (34/118) respectively did not recall that they had receive a medication-status-list at discharge. There was no significant difference between the groups ($p>0,05$).

Of all participants, 26 % (39/150) responded that they had questions regarding their medication after discharge (18% (6/33) in the control group and 28% (33/117) in the intervention group). Among those, 67 % in both the control group (4/6) and intervention group (22/33) found an answer to their question by searching the internet or contacting their general practitioner, pharmacy, hospital, relatives or visiting nurses. In total, 8 patients from the intervention group contacted the MIH and 100% (8/8) of those participants received an answer to their question.

Beliefs and perception of security about medicines

Both the control- and the intervention group responded that they felt safe to a great extent regarding their medication after discharge, and there was no significant difference between groups ($p=0.59$). Both groups expressed that their current health to some extent or to a great extent was dependent on medication. However, they did not worry about taking their medicine, nor did they

experience their medication as a mystery, and they expressed that the medication only disrupted their lives to a small extent (figure 3). No significant difference was found between the groups.

Figure 3 – Beliefs and perception of safety about medicine

Patients scored on a 5-point scale with 1=not at all and 5=to a very great extent. No significance was found between control- and intervention group for any of the questions (Mann-Whitney U test, $p>0.05$ for all questions).

Patient satisfaction with MIH

Participants in the intervention group were subsequently asked if the opportunity to contact the MIH affected the feeling of safety in relation to their medication, which majority of the participants acknowledged (72%, 79/110). Further, patients who contacted the MIH reported that the feeling of security increased after contacting the MIH, as they found the information reassuring and brought clarity to potential worries, such as side effects, dosage regimens etc. In addition, the patients informed that the helpline provided practical advice on how to administer the medication.

Only patients in contact with the MIH before the telephone interview were asked about their satisfaction with the service (n = 8). The participants contacted the MIH by phone and received an answer by phone. The participants found the answers provided by the MIH timely, easy to understand and relevant. Furthermore, they reported that the answers to a great extent affected their use of medicine in a positive manner, just as they were satisfied with the answers to a very great extent (4.71 ± 0.48 on a 5-point scale). Generally, the participants were satisfied with the service, they would use it again, and they believed the helpline should be a permanent service for discharged patients in the Capital Region of Denmark.

Clinical impact of MIH on patient safety

MRPs and the clinical impact of the helpline responses were assessed by two clinical pharmacists working at the MIC. All enquiries were included in the assessment regardless of when the patient asked the question relatively to the telephone interview, e.g. pre-, post- or during the interviews.

From the 37 enquiries, a total of 43 MRPs were identified according to the Westerlund system (figure 4). The most frequent MRP was uncertainty about the aim of the medicine, such as uncertainty on how to take insulin or whether two prescribed medications during hospitalization were the same and should both be administered similarly. Further, concerns about adverse reactions, interactions between medicines and therapy failure were identified MRPs in more than 10% of the enquiries (figure 4).

Figure 4 – Categorization of MRPs related to the enquiry according to the Westerlund system [15, 16].

The 43 MRPs extracted from 37 enquiries to the MIH. Other MRPs included enquiries about vitamins and the need to speak with either a hospital- or general physician. The categories without any MRPs were under-/overuse of medicine, difficulty opening the medicine container and inappropriate storage of the medicine (categories not displayed in figure).

The pharmacy interventions for each MRP were also identified using the Westerlund system. One MRP may trigger several pharmacy interventions; thus, a total of 77 were identified. Of these, medicine counselling was performed for the 43 MRPs extracted from the 37 enquiries to MIH. Further, the pharmacy interventions consisted of a referral to prescriber or other health-care provider (17 situations); provision of practical information (12 situations); direct contact from the pharmacy personnel with the hospital prescriber (3 situations), and provision of information to patients' representative (2 situations)

Clinical significance was assessed using the DOCUMENT system [17]. However, in most cases the clinical significance could not be attributed to a single specific pharmacy intervention. Instead it was a sum of several pharmacy interventions performed to alleviate the MRP. Thus, clinical assessment was performed at the level of each MRP. More than 50% of the MRPs resulted in pharmacy interventions assessed to have a high- or moderate clinical significance, among these were counselling on how to administer insulin; impact of concomitant treatment with ciprofloxacin and theophylline; and length of antibiotic treatment to a patient who had only received tablets for 3 days and no prescription (table 4).

Table 4 – Assessment of the clinical significance of the pharmacy interventions proposed to mitigate MRPs.

**Clinical significance**	**Number of MRPs (N=43)**
Nil (%)[a]	1 (2)
Low (%)[a]	8 (19)
Mild (%)[a]	11 (26)
Moderate (%)[a]	17 (39)
High (%)[a]	6[b] (14)

[a]Percentages of total number of MRP's

Discussion

The current study showed that patients discharged from hospital in general expressed a high level of satisfaction with the medicine information provided during hospitalization and at discharge. Despite this a considerable number of patients still had questions regarding their medication after hospital discharge. Patients offered access to contact a newly established hospital-based MIH showed a high level of satisfaction with the service provided by the MIH. Patients who were offered access to the helpline reported an increase in their feeling of security in relation to their medication. During the study period a total of 37 enquiries were received and answered. Analysis of the enquiries revealed 43 MRPs of which 53% triggered interventions assessed to have a moderate to high clinical significance. All patients in contact with the MIH were "to a great extent" satisfied with the MIH service[illegible]

Need for medicine information after discharge

The present study shows that patients have a need for information about their medication after discharge, more specifically, 26% of the participants in the current study. Similar results have been reported by Shuen et al. who found that 26% of patients discharged from an emergency department made a call or visit to their primary medical doctor or a specialist physician post discharge [20[illegible]In this study 8 out of 119 patients contacted the MIH after discharge. However, performing the telephone interviews (post discharge) triggered another 18 patients to make a total

of 37 enquiries. The fact that post discharge contact triggers medicine-related questions is not surprising and several studies have explored the impact of different follow-up strategies such as post-discharge home visits by pharmacist, follow-up phone calls and text messages in order to alleviate MRPs [11, 21, 22]. Although the number of patients taking a non-stimulated contact to the MIH is limited in this study, it should be seen in the context that the service is newly established to patients. As the MIH becomes a well-known service, we anticipate that the number of enquiries increase over time.

Increase patient safety

Analysis of the enquiries revealed that 53% triggered interventions assessed to have a moderate to high clinical significance, thus anticipating that the interventions improved the patient safety of the patient medication. It is well known that medication after discharge is indeed a major cause of rehospitalization [1-3]. Studies indicate that providing the appropriate level of medicine information after discharge may reduce medicine related rehospitalization or contact to primary medical doctor or physician [1, 4, 23, 24]. In this context a MIH may not only improve the patient safety of the medication after discharge but it may also decrease overall costs of treatment. However, further studies are needed to elucidate the cost-benefit of a MIH service to patients after discharge.

High level of patient satisfaction with MIH

This study showed a high level of satisfaction with the MIH. The participants found the answers to their enquiries comprehendible and relevant, which affected their use of medicine. Other medicine helplines have reported similar high ratings of user satisfaction where the majority of the patients report that they received sufficient information and felt reassured about their medicine [12]. In line with the current study, Badiani et al. found that 93% o[illegible] the respondents followed the advice provided in their study [7].

In this study the most common enquiries were related to dose, administration, effect and usage of medicines. The nature of these enquiries are similar to those found in the literature [5, 7, 12]. Further assessment of each enquiry revealed 43 MRPs, where the most frequent MRP was uncertainty about the aim of the medicine, which emphasize that the information given during hospitalization or at discharge was insufficient. Similar results have been reported from other medicine helplines where wrong or insufficient information about medicine is the main topic of enquiries [5, 8].

Perception of safety with medicine after discharge

Although patients in both groups reported that they to a high degree felt safe with their medication after discharge, the current study revealed that patients with access to the MIH expressed an increase in the feeling of security in relation to their medication. Many patients take action and seek information from e.g. friends, family or the internet in order to prevent harm [25] and here MIHs may indeed alleviate these worries and support and empower the patients to deal with the problems.

Relevant medical information not only enables the patients to prevent and solve MRPs, but it may also be a key factor in relation to adherence [22]. In this study adherence was not directly evaluated, however, the increase in feeling of security in relation to medication may have a positive effect on adherence. Future studies are needed to elucidate the role of MIH in relation to adherence.

Methodological considerations

The present study was using a randomized study design. The dropout rate in the current study was higher than anticipated (39%, 17 and 81 in the control and intervention group respectively) limiting the power to identify significant differences. A smaller limitation in the current study concerns the interview guide that includes some questions that are not fully validated. However, most of the questions in the interview guide were adopted word-for-word from other studies ensuring that the

majority of the questions had been tested by other researchers beforehand [7, 14]. Furthermore, the final interview guide was pilot tested thus assuring an acceptable level of validity. The study did not include the full Beliefs About Medicine Questionnaire [14], thus not allowing a fully valid conclusion on patients concerns and necessity of taking medicine.

A methodological consideration in the current study is the risk of respondent’s social desirability bias where respondents may response in a way they think the investigator wants to hear and hereby present themselves in the best possible way [26]. Social desirability bias may lead to a better rating of the medicine helpline service although neutral wordings were used in the questions and most questions were answered on a scale rather than single items.

Further, there is a risk of recall bias, which is a systematic error that occurs where the participants do not recall or remember previous experiences accurately or omit details related to the topic of interest [27, 28]. Since the interview in this study took place 2-4 weeks after discharge, there is a risk of recall bias, especially in regard to the medicine information during hospitalization and at discharge. However, recall bias is considered more prominent in retrospective surveys or self-reports, and the methodological approach in this study was telephone interviews where the researchers had the opportunity to probe and clarify the questions during the interviews. Thus, the extent of recall bias is considered minimal. Another limitation aspect is the fact that 40% of the included patients were lost in the follow-up and thus they were never interviewed. Although a 60% response rate is considered acceptable a high dropout rate might affect the generalizability of the study results

Participants in the current study were included from two departments, an emergency department and a department of respiratory medicine. Thus, the present results are most probably only representative of similar clinical settings. Indeed, future studies should investigate other clinical settings, such as departments for surgery or children.

Conclusion

MIH offers an important support for discharged patients to alleviate DRPs and increase the belief and perception of security with their medicine after discharge. This study emphasize that patients value the service and the information provided by the MIH, and that the service positively affects a safe medication after discharge. Changes in the medical treatment after discharge is challenging and patients should be provided with sufficient pharmaceutical support and information to overcome these struggles, ultimately ensuring a patient safe medication.

References

1. Williams, M., et al., *A systematic review examining the effectiveness of medicines information services for patients and the general public.* Int J Pharm Pract, 2020. **28**(1): p. 26-40.
2. Mackridge, A.J., et al., *Cross-sectional survey of patients' need for information and support with medicines after discharge from hospital.* Int J Pharm Pract, 2018. **26**(5): p. 433-441.
3. Daliri, S., et al., *The effect of a pharmacy-led transitional care program on medication-related problems post-discharge: A before-After prospective study.* PLoS One, 2019. **14**(3): p. e0213593.
4. Elson, R., H. Cook, and A. Blenkinsopp, *Patients' knowledge of new medicines after discharge from hospital: What are the effects of hospital-based discharge counseling and community-based medicines use reviews (MURs)?* Res Social Adm Pharm, 2017. **13**(3): p. 628-633.
5. Marvin, V., et al., *Phone calls to a hospital medicines information helpline: analysis of queries from members of the public and assessment of potential for harm from their medicines.* Int J Pharm Pract, 2011. **19**(2): p. 115-22.
6. Bramley, D., et al., *How useful are medicines helplines for patients discharged from hospital?* 2014. **292**.
7. Badiani, A., et al., *Impact of a medicines helpline for patients.* 2017. **24**(4): p. 196-199.
8. Bramley, D.M., et al., *The impact of Medicines Information enquiry answering on patient care and outcomes.* Int J Pharm Pract, 2013. **21**(6): p. 393-404.
9. Mitchell, B., C. Chong, and W.K. Lim, *Medication adherence 1 month after hospital discharge in medical inpatients.* Intern Med J, 2016. **46**(2): p. 185-92.
10. Pasina, L., et al., *Medication non-adherence among elderly patients newly discharged and receiving polypharmacy.* Drugs Aging, 2014. **31**(4): p. 283-9.
11. Sze, W.T., R. Pudney, and L. Wei, *Inpatients' satisfaction towards information received about medicines.* Eur J Hosp Pharm, 2020. **27**(5): p. 280-285.
12. Bramley, D., A. Innes, and N. Dass, *Impact of Medicines Helplines on patient satisfaction, patient outcomes and medicines safety for hospital patients: the development of a rating scale and an evaluation of patients' opinions.* Eur J Hosp Pharm, 2019. **26**(6): p. 301-307.
13. Region Hovedstaden, p. *MedicinInfo - lægemiddelrådgivning*. 2017 [cited 2019 13. february]; Available from: https://www.sundhed.dk/sundhedsfaglig/information-til-praksis/hovedstaden/almen-praksis/laegemidler/raadgivning/.
14. Horne, R., J. Weinman, and M. Hankins, *The beliefs about medicines questionnaire: The development and evaluation of a new method for assessing the cognitive representation of medication.* Psychology & Health, 1999. **14**(1): p. 1-24.
15. Westerlund, T. and B. Marklund, *Assessment of the clinical and economic outcomes of pharmacy interventions in drug-related problems.* J Clin Pharm Ther, 2009. **34**(3): p. 319-27.
16. Westerlund, L.T. and H.T. Björk, *Pharmaceutical care in community pharmacies: practice and research in Sweden.* Ann Pharmacother, 2006. **40**(6): p. 1162-9.
17. Williams, M., et al., *DOCUMENT: a system for classifying drug-related problems in community pharmacy.* Int J Clin Pharm, 2012. **34**(1): p. 43-52.
18. Park, H.Y., et al., *Medication adherence and beliefs about medication in elderly patients living alone with chronic diseases.* Patient Prefer Adherence, 2018. **12**: p. 175-181.
19. Neame, R. and A. Hammond, *Beliefs about medications: a questionnaire survey of people with rheumatoid arthritis.* Rheumatology (Oxford), 2005. **44**(6): p. 762-7.
20. Shuen, J.A., et al., *Telephoned, Texted, or Typed Out: A Randomized Trial of Physician-Patient Communication After Emergency Department Discharge.* J Emerg Med, 2018. **55**(4): p. 573-581.
21. Marvin, V., et al., *Medication-related problems after discharge from Acute Care: a telephone follow-up pilot survey.* European Journal of Hospital Pharmacy: Science and Practice, 2012. **19**: p. 112-112.
22. Horne, R. and J. Weinman, *Patients' beliefs about prescribed medicines and their role in adherence to treatment in chronic physical illness.* J Psychosom Res, 1999. **47**(6): p. 555-67.
23. Oscalices, M.I.L., et al., *Discharge guidance and telephone follow-up in the therapeutic adherence of heart failure: randomized clinical trial.* Rev Lat Am Enfermagem, 2019. **27**: p. e3159.
24. Health Quality Ontario, *Effects of early follow-up after hospital discharge on outcomes in patients with heart failure or OCP disease*, in *ONTARIO HEALTH TECHNOLOGY ASSESSMENT SERIES*, J. Song and M. Walter, Editors. 2017: Health Quality Ontario. p. 37.

25. Fylan, B., et al., *A qualitative study of patient involvement in medicines management after hospital discharge: an under-recognised source of systems resilience.* BMJ Qual Saf, 2018. **27**(7): p. 539-546.
26. Bowling, A., *Research Methods in Health: investigating health and health services*. 4 ed. 2014, Maidenhead, GB: McGraw Hill, open University Press. 536.
27. Henriksen, K. and H. Kaplan, *Hindsight bias, outcome knowledge and adaptive learning.* Qual Saf Health Care 2003. **12**: p. ii46–ii50.
28. McCambridge, J., J. Witton, and D.R. Elbourne, *Systematic review of the Hawthorne effect: new concepts are needed to study research participation effects.* J Clin Epidemiol, 2014. **67**(3): p. 267-77.

Figure 1 Click here to access/download;Figure;Figure 1.docx

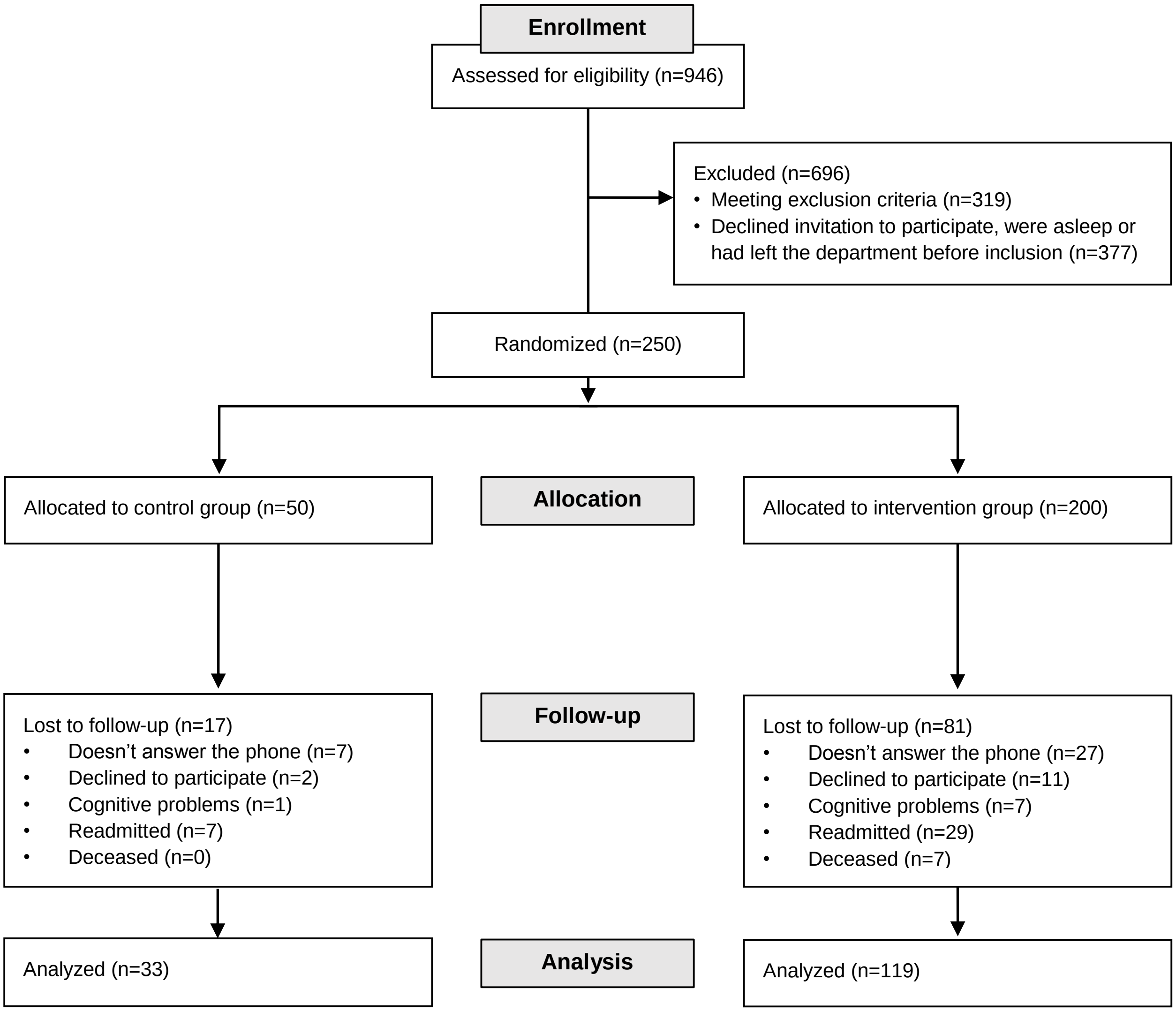

Figure 2

Click here to access/download;Figure;Figure 2.docx

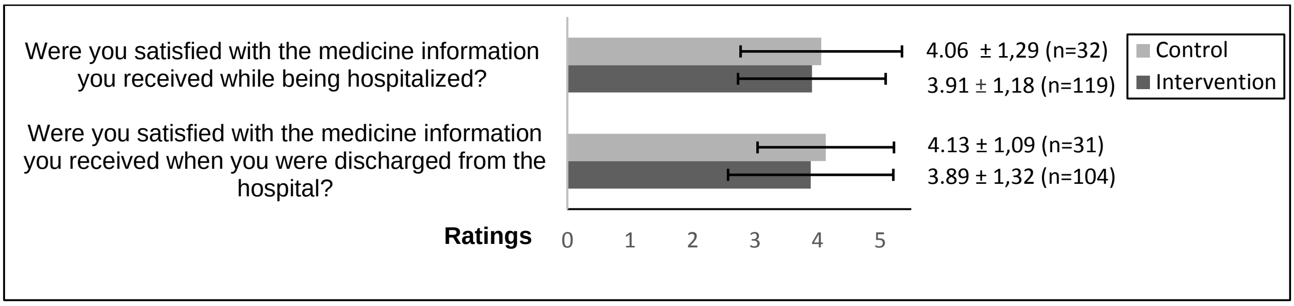

Figure 3

Click here to access/download;Figure;Figure 3.docx

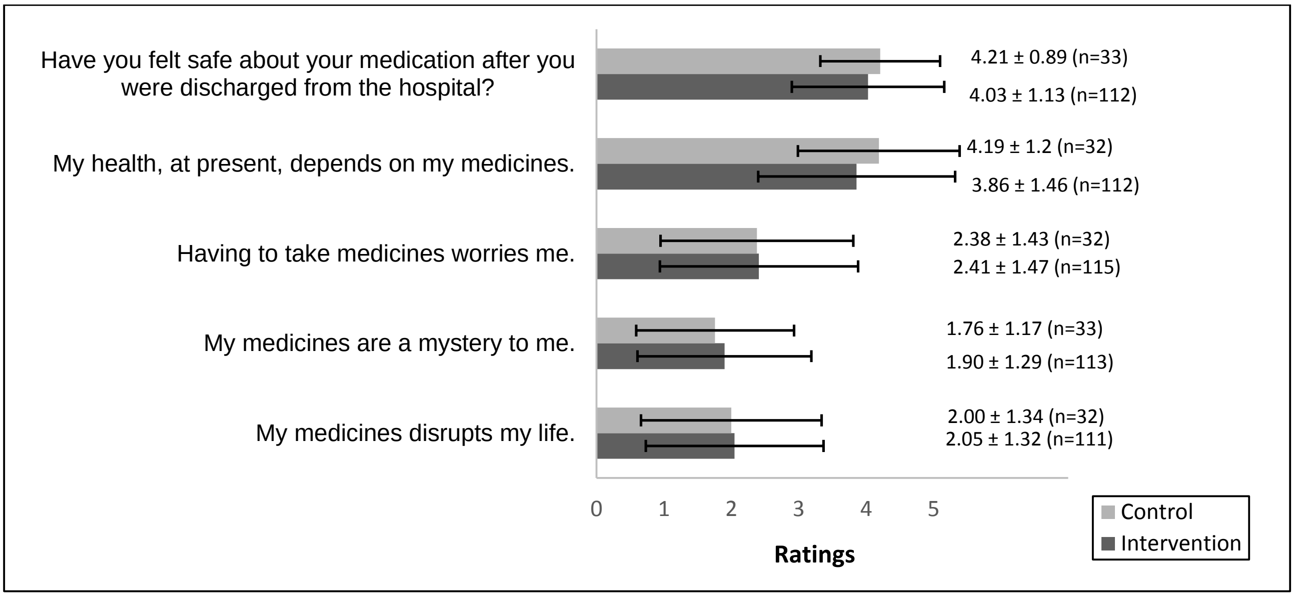

Figure 4

Click here to access/download;Figure;Figure 4.docx

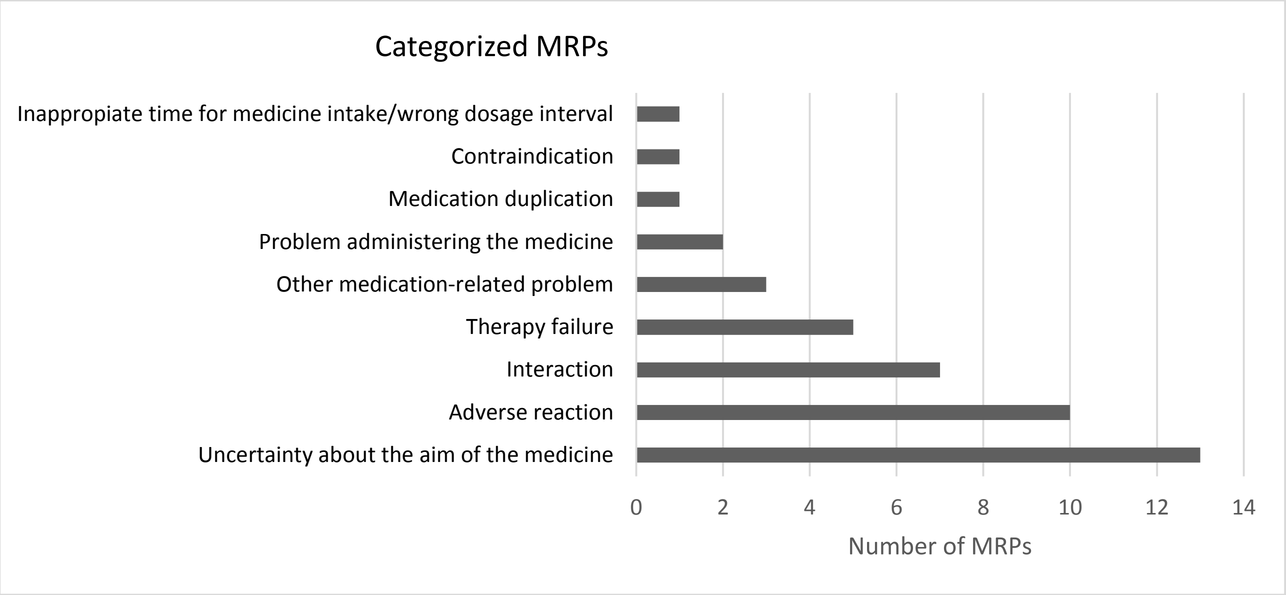

Click here to access/download
Supporting Information
S1 CONSORT Checklist.doc

Click here to access/download
Supporting Information
S2 Appendix.docx

Click here to access/download
Supporting Information
S3 Appendix.docx

Supporting Information S4

Click here to access/download
Supporting Information
S4 Ethic committee protocol_danish.docx

Click here to access/download
Supporting Information
S5 Ethics Committee protocol_english.docx
