# Supplementary material for: Medicine information helpline after hospitalization–a randomized trial: Impact on patient satisfaction, patient concerns about medicines and clinical outcome on patient safety
Source: PLoS One. 2023 Oct 26;18(10):e0293523. doi: 10.1371/journal.pone.0293523 (PMC10602279; doi:10.1371/journal.pone.0293523)
Supplement: Attachment — Submitted filename: Response to Reviewers.docx [file pone.0293523.s010.docx]

Copenhagen, September 14^th^ 2023

Rebuttal letter: Medicine Information Helpline after hospitalization – a randomized trial: impact on patient satisfaction, patient concerns about medicines and clinical outcome on patient safety

Dear Dr. Vineet Gupta and reviewers

Thank you for giving us the opportunity to revise the manuscript. We thank you for your review which have given us a chance to further improve the manuscript. In the following we have addressed all the concerns raised and we have done our best to revies the manuscript accordingly. We hope that this revision is acceptable to your standards and that you find the manuscript ready for publication in PLOS ONE.

Yours sincerely,

Marianne Hald Clemmensen

Head of Clinical Pharmacy, Royal Hospital of Denmark

Rigshospitalet og Bornholm

Esther Møllers Vej 2

2100 Copenhagen Ø

Denmark

**Changes to reference list**

There have been no changes to the reference list. Thus, the submitted reference list is identical to the one submitted previously.

**Reviewer 2:**

1- Sample size estimate: State the statistical testing method which achieves 90% power.

*No statistical testing method was used to achieve a 90% power. The power was set by the authors to 90% which we consider to be a conservative choise as most studies will accept a power of 80%. A small clarification has been added to the data analysis to clarify that the level of power was set by the authors (line 193-194).*

2- Define SD at its first appearance.

*Thank you for the comment. We have added an explanation to “SD” when it is first introduced in the manuscript (line 202).*

3- The Wilcoxon rank sum test is the complete name of the test.

*The manuscript has been updated accordingly.*

4- Table 2: Remove repeat p-values for individual characteristics. For instance, place the p-value of 0.540 on the line for "Sex, No. (%)" rather than on the lines for Male and Female. Also relabel this characteristic "Gender".

*We thank the reviewer for the comment and has updated Table 2 as suggested.*

5- Figure 2 caption: Clarify that "No significance" refers to "No significant differences".

*The Figure 2 caption has been revised as suggested.*

6- In the "Data analysis" section, state the level of the p-value that has been used to define statistically significant differences. For example many publications include the following sentence: P-values less than 0.05 were considered statistically significant.

*The manuscript has been updated with the suggested wording in the Data analysis section (line 193-194).*

7- Patient satisfaction with MIH: The first sentence of this paragraph suffers from subject-verb disagreement. The corrected sentence reads, "One of the primary outcomes was patient satisfaction with MIH."

*Thank you for this review. The manuscript has been updated accordingly.*

8- For primary outcomes reported as percentages, provide 95% confidence intervals for these percentages. State the statistical method used to estimate these confidence intervals in the "Data analysis" section of the manuscript.

*The primary outcomes are reported as follows:*

- *Patient satisfaction with the MIH is reported as a mean score and the CI can be found in S7 Supporting Information, Question 25.*
- *Patients feeling of safety regarding their medication is assessed by Question 9-14. Question 9-13 is reported as mean score and the CI can be found in S7 Supporting Information. Question 14 is reported as percentage and a confidence interval has been added to the manuscript regarding question 14 (line 289). Furthermore, a sentence has been added to the method section describing the method used to calculate the CI (line 203).*
- *Clinical impact of MIH is reported with exact numbers (descriptive statistics) and thus a CI is not calculated.*

9- Discussion: Subject-verb disagreement correction: "As expected, the rate of patients who had a question regarding their medicines was higher ..."

*Thank you for the input. The manuscript has been corrected.*

10- To assist in the review process, add line numbering to the document.

*Line numbering has been added to the document.*
